# Vangl2-Regulated Polarisation of Second Heart Field-Derived Cells Is Required for Outflow Tract Lengthening during Cardiac Development

**DOI:** 10.1371/journal.pgen.1004871

**Published:** 2014-12-18

**Authors:** Simon A. Ramsbottom, Vipul Sharma, Hong Jun Rhee, Lorraine Eley, Helen M. Phillips, Hannah F. Rigby, Charlotte Dean, Bill Chaudhry, Deborah J. Henderson

**Affiliations:** 1 Institute of Genetic Medicine, Newcastle University, Centre for Life, Newcastle upon Tyne, United Kingdom; 2 Leukocyte Biology, National Heart and Lung Institute, Imperial College London, London, United Kingdom; 3 Mammalian Genetics Unit, MRC Harwell, Oxfordshire, United Kingdom; Harvard Medical School, United States of America

## Abstract

Planar cell polarity (PCP) is the mechanism by which cells orient themselves in the plane of an epithelium or during directed cell migration, and is regulated by a highly conserved signalling pathway. Mutations in the PCP gene *Vangl2*, as well as in other key components of the pathway, cause a spectrum of cardiac outflow tract defects. However, it is unclear why cells within the mesodermal heart tissue require PCP signalling. Using a new conditionally floxed allele we show that Vangl2 is required solely within the second heart field (SHF) to direct normal outflow tract lengthening, a process that is required for septation and normal alignment of the aorta and pulmonary trunk with the ventricular chambers. Analysis of a range of markers of polarised epithelial tissues showed that in the normal heart, undifferentiated SHF cells move from the dorsal pericardial wall into the distal outflow tract where they acquire an epithelial phenotype, before moving proximally where they differentiate into cardiomyocytes. Thus there is a transition zone in the distal outflow tract where SHF cells become more polarised, turn off progenitor markers and start to differentiate to cardiomyocytes. Membrane-bound Vangl2 marks the proximal extent of this transition zone and in the absence of Vangl2, the SHF-derived cells are abnormally polarised and disorganised. The consequent thickening, rather than lengthening, of the outflow wall leads to a shortened outflow tract. Premature down regulation of the SHF-progenitor marker Isl1 in the mutants, and accompanied premature differentiation to cardiomyocytes, suggests that the organisation of the cells within the transition zone is important for maintaining the undifferentiated phenotype. Thus, Vangl2-regulated polarisation and subsequent acquisition of an epithelial phenotype is essential to lengthen the tubular outflow vessel, a process that is essential for on-going cardiac morphogenesis.

## Author Summary

Congenital heart defects are common, affecting almost 1% of all live births. Many of these affect the outflow region, where the aorta and pulmonary trunk connect with the main ventricular chambers. Congenital heart defects arise from disruption of normal developmental processes and can be modelled in mice. Thus, studying normal development, together with mouse mutants that develop heart malformations, should shed light on why these common anomalies arise. We have studied cardiac development in a mouse mutant for the *Vangl2* gene, a key component of the planar cell polarity (PCP) pathway. This pathway controls the orientations of cells in epithelia and during directional cell migration. Here, we show that PCP signalling is required by cells derived from the second heart field, which forms the outflow tract walls. We show that in the absence of Vangl2, the cells within the distal outflow tract walls are non-polarised and disorganised. As a consequence the outflow tract is shortened and does not align properly with the ventricles. Thus, we show why disruption of a key PCP gene leads to outflow tract malformations. This is important for understanding heart development, but also more generally for understanding how PCP signalling regulates growth of tubular structures.

## Introduction

Malformations affecting the outflow of the heart are a major cause of morbidity and mortality in childhood. While many of these malformations occur sporadically, studies of families with congenital heart defects, alongside animal studies, have revealed that phenotypically discrete heart malformations can have diverse causes. These can involve disruption of a number of different genes, embryonic lineages or developmental processes. Furthermore, dissimilar malformations, including double outlet right ventricle, common arterial trunk, and tetralogy of Fallot, may appear in offspring sharing the same genetic defect and can therefore be considered within a spectrum of malformation with similar underlying causes [1]. Clarifying the fundamental processes that underpin cardiovascular development is essential to understand this complexity.

The primitive heart tube is derived from the cardiac crescent, or first heart field, at embryonic day (E) 8.5 of mouse development. Subsequently, the second heart field (SHF), which lies dorso-anteriorly to the primary cardiac crescent, adds cells to both the venous (inflow) and arterial (outflow) poles to lengthen the primitive heart tube [2], [3], [4]. The outflow tract develops as a bi-layered tube composed of an outer layer of myocardium, with an inner endocardial lining, both derived from the SHF [5]. This is connected proximally to the common ventricle and to the developing pharyngeal arch arteries at its distal end. Studies in chicken have shown that there is a focus of proliferative cells in the dorsal pericardial wall that act as a source of cells for both poles of the heart [6]. Moreover, these studies support the idea that the cells move into the outflow as an epithelial sheet, rather than as individually migrating cells. Although the precise morphogenetic mechanisms underpinning outflow development are still being elucidated, the targeted disruption of a number of genes within the SHF, including *Isl1, Fgf8, Tbx1*, and *Tbx20*, give rise to outflow tract malformations in mice (reviewed in [7]). Furthermore, mutations in *TBX1* [8] and common variants in *ISL1* [9] have been found in human patients with outflow tract malformations, indicating a developmentally conserved role for the SHF during cardiac development. Detailed studies have shown that the transcriptional network involving *Tbx1* and *Isl1* is required to maintain SHF cells in a proliferative, progenitor-like state before they are added to the outflow tract [10]; in their absence the outflow tract is shortened. Following outflow tract septation, this results in mal-alignment of the aorta and pulmonary trunk with the ventricular chambers [5], [11]. Despite insight into the transcriptional network that regulates the maintenance of SHF progenitors before they reach the poles of the heart, there is limited information about the characteristics and behaviour of the cells as they add to the outflow tract.

Endocardial cushions form along the length of the developing outflow tract and by way of complex processes of cell migration, growth and remodelling, result in the separation of the initially single vessel into the aortic and pulmonary trunks (reviewed in [12]). Neural crest cells (NCC), originating in the cranial neural tube, migrate long distances into the pharyngeal arches and endocardial cushions and are involved in septation and alignment of the outflow vessels [13], [14], [15]. The processes regulating NCC migration have been studied in some detail, and at least in frogs and zebrafish, contact inhibition of locomotion, regulated at least in part by the planar cell polarity signalling pathway (see below) is implicated [16], [17]. Thus, a variety of cell types and complex morphological processes contribute to the developing outflow tract.

The planar cell polarity (PCP) pathway is a non-canonical Wnt pathway, which acts to regulate cell polarity within the plane of an epithelium. Studies in *Drosophila* wing, eye and abdomen have shown that polarity between adjacent cells is co-ordinated by the asymmetrical localisation of core PCP factors. Vangl2 (Strabismus), Flamingo and Prickle accumulate proximally in polarised cells, while Dishevelled, Frizzled, Flamingo, and Diversin (Diego) accumulate distally [18]. PCP signalling has also been implicated in the regulation of apico-basal polarity, and directional cell migration [19], [20], [21]. Outflow tract malformations, including septation defects, are common in mice following the disruption of PCP genes (reviewed in [22]), although the lineage requirement for PCP signalling in heart development remains unclear. *Loop-tail* (*Lp*) mice carry a mutation in the *Vangl2* gene, encoding a key component of the PCP pathway. *Lp/Lp* mice display a number of malformations associated with disrupted PCP signalling, including misorientation of stereocilia in the cochlea and utricle [23], [24] and craniorachischisis [25]. We have previously shown that *Lp/Lp* embryos have a spectrum of cardiac defects that affect the outflow region of the heart [26], including double outlet right ventricle, common arterial trunk, abnormal patterning of the pharyngeal arches and ventricular septal defects. The mutant embryos also have abnormalities in the ventricular wall that include the coronary arteries [27]. Thus, the spectrum of defects seen in *Lp/Lp* mice could result from disruption of several cell types. Here we investigate the role of Vangl2 during outflow tract development using a novel tissue-specific knockout of the *Vangl2* gene. We highlight a role for Vangl2 specifically within the SHF and show that Vangl2 is essential for forming the epithelial distal component of the elongating outflow vessel.

## Results

### Spectrum of outflow abnormalities in *Lp* mice

**Figure 1:**
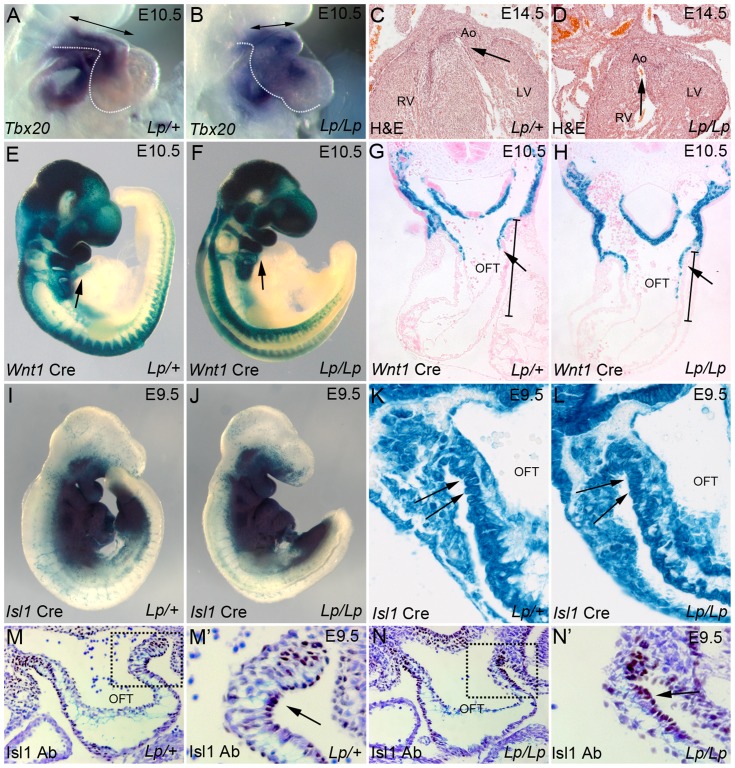
*Lp* mice display a spectrum of outflow tract abnormalities. **A,B**) *In situ* hybridisation on E10.5 *Lp/+* and *Lp/Lp* embryos reveals normal expression of *Tbx20* in the mutant embryo, but illustrates the abnormal heart loop (the outline of the outflow tract and ventricular chambers is indicated by the dotted lines). **C,D**) H&E sections of E14.5 *Lp/+* and *Lp/Lp* embryos show the double outlet right ventricle in the mutant embryo (the arrows indicate the communication between and the aorta and the ventricle). **E–H**) β-gal staining (blue) of wholemount stained *Lp/+* and *Lp/Lp* E10.5 embryos shows that NCC migration (labelled by *Wnt1-Cre* based lineage tracing) appears normal in the mutants. Transverse sections (G,H) show that although the OFT is reduced in length, there is normal migration of NCC into the outflow vessel (arrow). The bars in G,H indicate the characteristic shortened outflow tract seen in the mutant. **I–L**) β-gal staining of wholemount stained *Lp/+* and *Lp/Lp* E9.5 embryos shows that the SHF, labelled by *Isl1-Cre* based lineage tracing, appears normal in the mutants, however the cells appear disorganised (arrows). **M,N**) Isl1 antibody labels SHF cells in the distal outflow tract (brown staining – arrows). These cells appear disorganised in the *Lp/Lp* embryo at E9.5 (N′ arrow, compare to M′). Ao – aorta, LV - left ventricle, OFT - outflow tract, RV - right ventricle.

We have shown previously that *Lp/Lp* embryos present a number of cardiac anomalies [26]. To begin to characterise early heart formation in these mutants and as a prelude to lineage-based analyses, cardiac-specific markers were analysed by *in situ* hybridization, in order to determine whether the chambers formed properly. The expression patterns of the chamber markers *Mlc2a*, *Mlc2v* and *Nppa*, and outflow markers including *Tbx20*, were examined in embryos from *Lp* litters at E10.5. Whilst none of these markers showed reproducible expression differences between control and *Lp/Lp* embryos (n = 2–3 for each gene examined), aberrant heart looping could clearly be seen in the latter; the outflow tract was shorter and the right ventricle was hypoplastic, although to a varying extent, in all *Lp/Lp* when compared with controls (>50 embryos examined in total; Fig. 1 A,B, S1 Fig.). Transverse sections of *Lp/Lp* hearts at E14.5 revealed double outlet right ventricle (Fig. 1 C,D) as previously described [26]. To begin to establish the cell type that requires Vangl2 signalling for outflow tract development, we first crossed the *Lp* mice with the *Wnt1-Cre* line. However, no defects in NCC migration were observed in *Lp/Lp* embryos from E10.5, with the distribution of *Wnt1-Cre*-positive cells indistinguishable from that of control littermates (Fig. 1 E–H ).

### Disorganised movement of Isl1-positive cells into the outflow tract in *Lp/Lp*

Isl1 is expressed by all SHF progenitor cells and thus can be used for lineage tracing of the SHF [5]. We therefore asked whether *Isl1-Cre*-expressing SHF derivatives contribute normally to the heart in *Lp/Lp* embryos. Comparison with stage-matched controls revealed no abnormalities in the overall distribution of SHF-derivatives in the pharyngeal and cardiac regions of the *Lp/Lp* embryos (Fig. 1 I–L). As development progresses, the continued expression of Isl1 protein is confined to non-differentiated SHF progenitors; it is down-regulated as they differentiate [5]. At E9.5, Isl1-expressing cells localised to the mesenchyme of the dorsal wall of the aortic sac and the distal outflow tract (Fig. 1M). Whilst Isl1 staining was broadly similar between wild type and *Lp/Lp* littermates, Isl1-expressing cells appeared disorganised in the distal outflow tract in the mutant embryos (Fig. 1 compare N′ with M′). This subtle anomaly was highly reproducible (n = 10). Together, these data suggested that there could be an abnormality in SHF-derived cells in the distal outflow tract.

### Global loss of Vangl2 using *Vangl2^flox^* mice recapitulates the *Lp/Lp* phenotype

**Figure 2:**
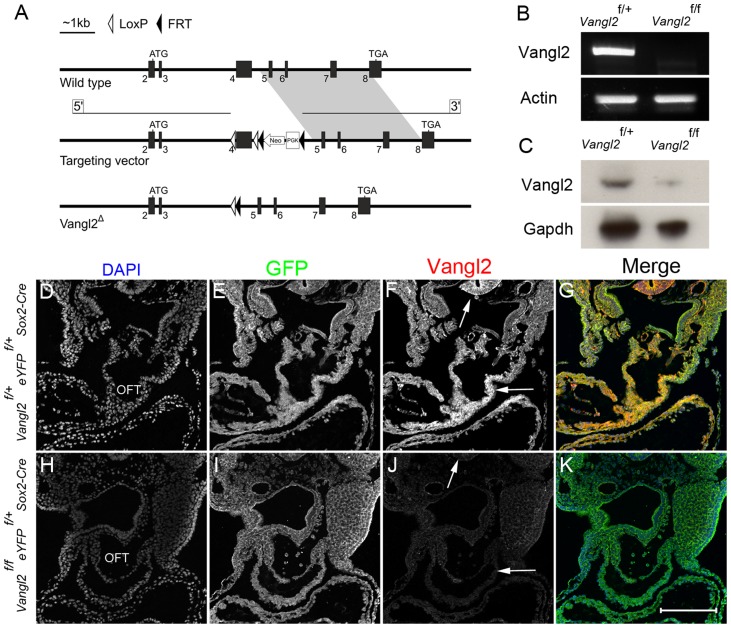
Targeting strategy and confirmation of knockdown. **A**) Cartoon indicating the targeting strategy. Disruption of the *Vangl2* gene was achieved by modification of the wild type allele to insert *LoxP* sites flanking exon 4. Expression of *Cre* recombinase results in the excision of exon 4 and subsequent loss of the transmembrane domains. **B**) RT-PCR on RNA isolated from whole E10.5 *Vangl2^flox/flox^; Sox2-Cre* embryos showed that there was no *Vangl2* transcript produced in the mutants, although this was abundant in controls. *Actin* was used as a loading control. **C**) Western blotting using protein isolated from whole E15.5 *Vangl2^flox/flox^; Sox2-Cre* embryos showed that there was a major reduction in Vangl2 protein in the mutant embryos, although the presence of a faint band suggested that the *Cre* was not 100% efficient at later stages. Gapdh was used as a loading control. **D–K**) Immunohistochemistry for *Sox2-Cre* (using eYFP as a reporter for *Cre* expression) showed that recombination was variable across the embryo in the mutants (E,I). However, immuno-staining for Vangl2 showed that the protein was lost from the outflow tract (J, compare to F; in F strong staining is apparent within the OFT and neural tube - arrows). Also see S2 Fig. OFT - outflow tract, *Vangl2^f^* – *Vangl2^flox^*. Scale bar  = 200 µm.

The gross morphological defects, including craniorachischisis and axial rotation defects, together with the loss of Vangl2 in all body cells, limit the use of *Lp* for studying the causes of the cardiac malformations. To clarify the role that Vangl2 plays during heart morphogenesis, we produced a floxed allele of *Vangl2*, in which exon 4, encoding the trans-membrane domains, is flanked by *loxP* sites (Fig. 2A). A *neomycin* selection cassette, flanked by *FRT* sites, was placed downstream of exon 4. *Vangl2^neo/neo^* mice, which retain the *neomycin* selection cassette within the construct, were hypomorphic for *Vangl2*, with 2/3 displaying craniorachischisis and 1/3 spina bifida only (S2 A–H Fig.). The *neoR* cassette was subsequently removed by crossing the *Vangl2^neo^* mice with *FLPe* mice, to give *Vangl2^flox^* mice. Recombination of the resulting construct following the expression of *Cre* is predicted to produce a premature stop codon that gives rise to a small 8 KDa protein, which lacks the four trans-membrane domains and C-terminal PDZ-binding domain required for interaction of Vangl2 with other proteins [28], [29].

*Vangl2^flox^* mice were crossed with *Sox2-Cre* and *PGK-Cre* mice to produce embryos containing the truncated *Vangl2* construct in all cells (Fig. 2 B–K). Inter-crossing *Vangl^flox/+^; Sox2-Cre* or *Vangl2^flox/+^; PGK-Cre* mice with *Lp/+* mice generated *Vangl2^flox/Lp^; Sox2-Cre* and *Vangl2^flox/Lp^; PGK-Cre* embryos that were indistinguishable from stage-matched *Lp/Lp* embryos, showing craniorachischisis and heart malformations (n = 4; S2 I–P Fig.). Thus, *Lp* and the *Vangl2* deletion allele failed to complement.

**Figure 3:**
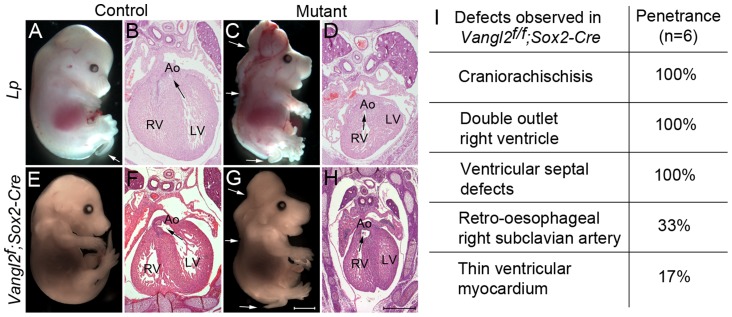
Global loss of Vangl2 recapitulates the *Lp/Lp* phenotype. **A–D**) At E14.5, *Lp/Lp* embryos exhibit gross abnormalities in body patterning including the severe neural tube defect craniorachischisis (arrows in C). Sectioning of these embryos revealed double outlet right ventricle (the arrows show the communication between the ventricle and the aorta). **E–H**) The phenotype of the *Vangl2^flox/flox^; Sox2-Cre* embryos (G,H) was indistinguishable from *Lp/Lp* (C,D). The *Vangl2^f/+^; Sox2-Cre* however did not have a looped tail, whereas this can be seen in *Lp/+* embryos (compare E with A white arrow). **I**) Breakdown of the cardiac defects seen in the *Vangl2^flox/flox^; Sox2-Cre* embryos at E14.5. Also see S3 Fig. Ao – aorta, LV - left ventricle, RV - right ventricle, *Vangl2^f^* – *Vangl2^flox^*. Scale bar  = 2 mm (white), 500 µm (black).

We next asked whether global knockout of *Vangl2* with *Sox2-Cre* recapitulated the *Lp/Lp* phenotype (Fig. 3 A–H). Indeed, *Vangl2^flox/flox^; Sox2-Cre* embryos had a shortened body axis and craniorachischisis (Fig. 3 compare G with C). Sectioning at E14.5 confirmed that the *Vangl2^flox/flox^; Sox2-Cre* embryos had heart malformations, including double outlet right ventricle, ventricular septal defects and pharyngeal arch remodelling defects, as are seen in *Lp/Lp* embryos (Fig. 3H and S3 A,B Fig. compare with Fig. 3D and [26]). Similar results were obtained using the *PGK-Cre* line in place of *Sox2-Cre* (S3 E–H Fig.). Thus, our novel *Vangl2^flox^* allele, when knocked out globally, recapitulated the phenotypes observed in *Lp/Lp* mutants and consequently was a potentially useful tool for genetically dissecting the *Lp/Lp* phenotype.

### Specific deletion of *Vangl2* within the SHF recapitulates the *Lp/Lp* outflow phenotype

**Figure 4:**
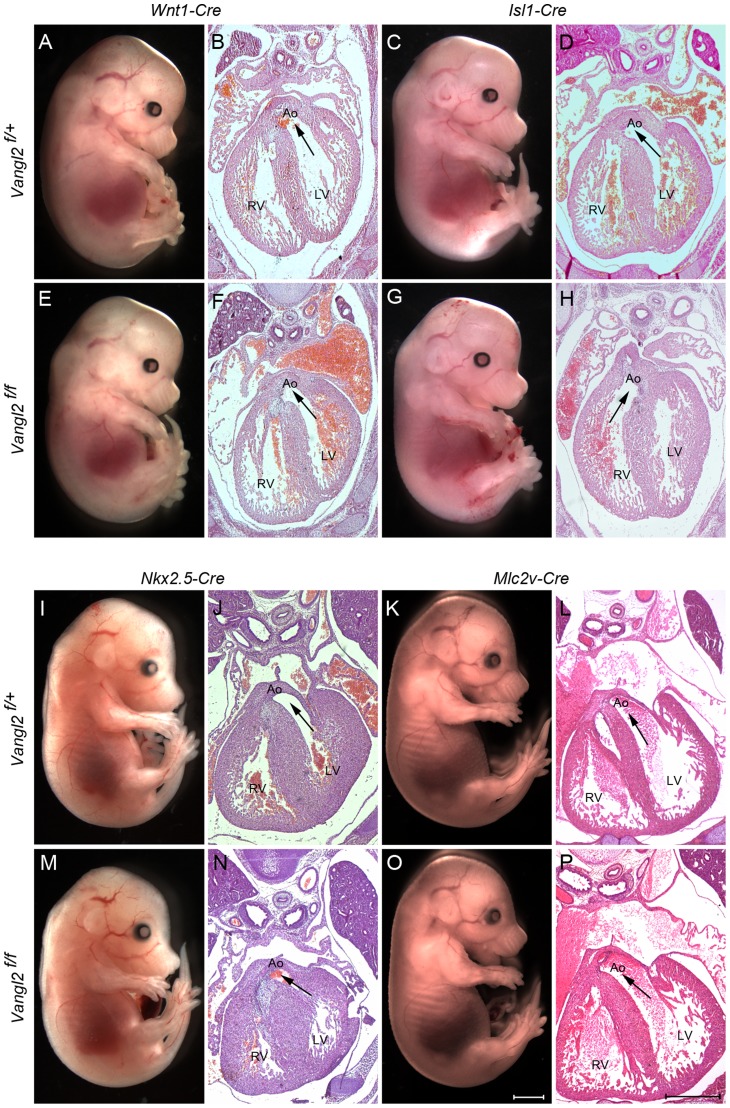
SHF-specific loss of *Vangl2* results in outflow tract defects. **A,B,E,F**) Targeted deletion of Vangl2 by *Wnt1-Cre*, in NCC, does not result in neural tube (A,E) or outflow tract defects (B,F). **C,D,G,H**) In contrast, although there are no neural tube defects when *Vangl2* is deleted in the *Isl1-Cre* expressing SHF (G), the resultant embryos do have double outlet right ventricle (H – compare with D). **I–P**) No defects were seen when *Vangl2* was deleted in either *Nkx2.5-Cre* expressing cardiac progenitors or *Mlc2v-Cre* expressing differentiated cardiomyocytes. In each case the arrows show the communication between the ventricle and the aorta. All embryos are E14.5. Also see S4 Fig. Ao – aorta, LV - left ventricle, RV - right ventricle, *Vangl2^f^* – *Vangl2^flox^*. Scale bar  = 2 mm (white), 500 µm (black).

In order to determine the tissue-specific requirement for *Vangl2* during heart development, we used a number of lineage-specific *Cre* driver lines to delete *Vangl2* in a targeted manner. Although our studies in *Lp/Lp* had suggested that NCC deficiency was unlikely to be the cause of the outflow defects, we could not rule out more subtle defects in their function. Therefore, to exclude the possibility that *Vangl2* is required in NCC for outflow tract development, we inter-crossed *Vangl2^flox^* mice with the *Wnt1-Cre* line. *Vangl2^flox/flox^; Wnt1-Cre* embryos were indistinguishable from control littermates with both a normal external appearance and normal hearts at E14.5 (n = 6; Fig. 4 A,B,E,F). Moreover, *Vangl2^flox/flox^; Wnt1-Cre* animals (n = 3) were viable and indistinguishable from their control littermates at 28 days after birth. Indeed, close analysis of the expression pattern of *Wnt1-Cre* compared with that of Vangl2 suggested that Vangl2 is not expressed by NCC, and that there was no change in the expression pattern of Vangl2 in the *Vangl2^flox/flox^; Wnt1-Cre* embryos at least at the stages when NCC are migrating into the heart (S4 Fig.). Thus, Vangl2 does not appear to be required by NCC for normal development of the outflow tract of the heart.

In order to test directly our hypothesis that Vangl2 is required in the SHF, we inter-crossed the *Vangl2^flox^* mice with *Isl1-Cre* and confirmed loss of Vangl2 in the outflow tract by immunofluorescence at E9.5 (S5 A–I Fig.). In contrast, Vangl2 expression was maintained outside the *Isl1-Cre* expression domain (S5I Fig.). While externally, the E14.5 *Vangl2^flox/flox^; Isl1-Cre* embryos were indistinguishable from their control littermates (Fig. 4 C,G), histological sectioning revealed double outlet right ventricle in 14/15 of the mutant embryos. Moreover, they all had a sub-aortic ventricular septal defect (Fig. 4 D,H and S5 N–S Fig.). An abnormality in the myocardialisation of the outflow cushions was also observed in *Vangl2^flox/flox^; Isl1-Cre* embryos (S6 Fig.), as was seen in *Lp/Lp* [30]. However, there were no abnormalities in pharyngeal arch patterning or the ventricular wall (S5 Fig.). Subsequent analysis at earlier stages (E9.5–E10.5) showed that the embryos had a markedly shortened outflow tract (S5 J–M Fig.). These data exclude the possibility that the outflow tract anomalies are secondary to the gross abnormalities in body patterning seen in *Lp/Lp*. They do, however, support the idea that *Vangl2* plays a specific role in the SHF.

We asked whether the role of *Vangl2* is restricted to the SHF or might play a more general role in cardiac progenitor populations. To test this idea, we crossed the *Vangl2^flox^* mice with *Nkx2.5-Cre* mice; *Nkx2.5-Cre* is expressed throughout the primitive heart tube but also in the cells derived from the SHF [31], [32]. Surprisingly, analysis of *Vangl2^flox/flox^; Nkx2.5-Cre* embryos at E15.5 revealed no obvious structural cardiovascular abnormalities (n = 4; Fig. 4 I,J,M,N). Our analysis of the *Nkx2.5-Cre* expression pattern largely confirmed previous reports, although we observed patchy expression in the outflow tract, compared with much broader and higher level expression in the left ventricle and atria (S7 Fig.). Moreover, Vangl2 was maintained in the distal outflow of *Vangl2^flox/flox^; Nkx2.5-Cre* embryos at E9.0 (S7 Fig.). This suggests that *Nkx2.5-Cre*, at least in our hands, may not be driving high enough levels of *Cre* to fully delete *Vangl2* in the outflow tract.

**Figure 5:**
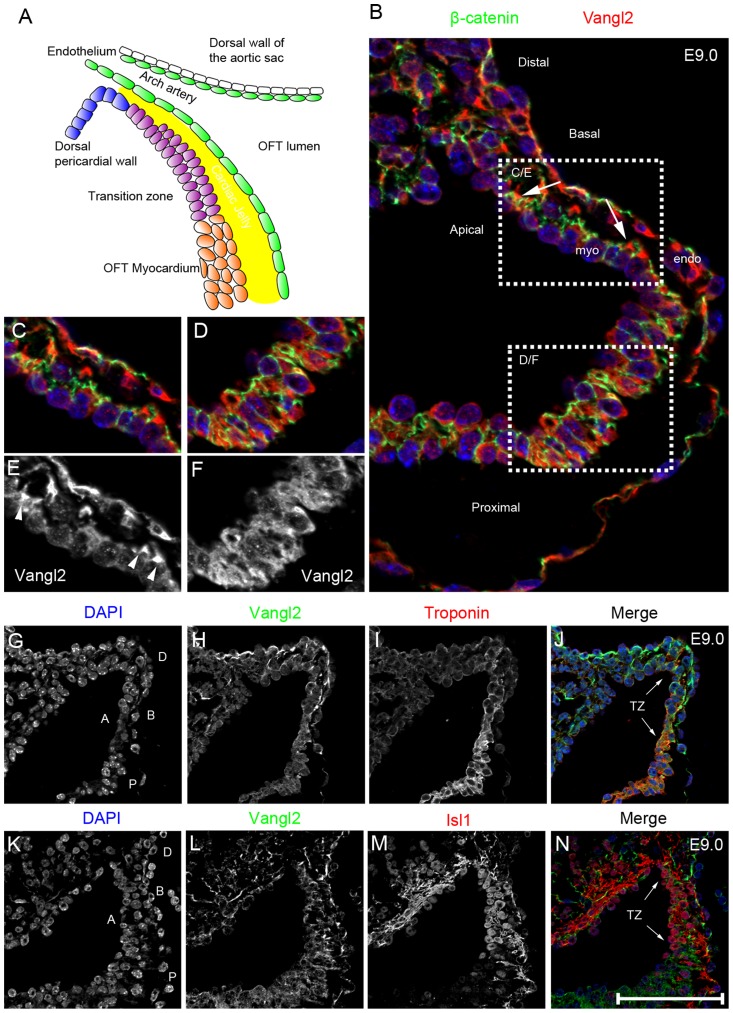
Vangl2 is expressed in the distal outflow region. **A**) Cartoon showing the region encompassing the dorsal pericardial wall and the distal outflow tract, including the region we describe as the transition zone. **B–F**) Vangl2 protein (red), labelled by immunofluorescence, is expressed in the distal outflow region (B), localising to the basal part of the membrane of the cells (as shown by co-localisation with β-catenin, a baso-lateral marker; green) in the dorsal pericardial wall and transition zone (B,C,E), but is found diffusely in the cytoplasm more proximally (B,D,F). **G–H**) Cardiac troponin I staining (red; labelling cardiomyocytes) is initially weak within the distal outflow but is upregulated more proximally (I). Vangl2 (green) and cardiac troponin I are co-expressed in the transition zone (J - TZ and arrows) of the outflow tract with the membrane-localisation of Vangl2 gradually lost (H) as cardiac troponin I staining becomes stronger. **K–N**) Vangl2 and Isl1 are also co-expressed in the cells of the transition zone (N - TZ and arrows), with the loss of Vangl2 from the membrane proximally coinciding with the loss of nuclear Isl1 localisation (N - lower white arrowhead). All images shown are of Vangl2^f/+^ embryos. A =  Apical, B =  Basal, D =  distal, endo  =  endocardium, myo  =  myocardium, P =  proximal, TZ =  transition zone. Scale bar  = 25 µm.

Finally, as we had previously shown abnormalities in the outflow tract myocardium in *Lp* mutants [30], and because *Isl1-Cre* also drives expression in endocardial cells in the outflow tract (Fig. 5B), we wanted to establish whether *Vangl2* is required in differentiated cardiomyocytes or the endocardium. To investigate this, we inter-crossed the *Vangl2^flox^* mice with *Mlc2v-Cre* mice, to drive *Cre* in the outflow and ventricular myocardium. At E9.5–E10.5, *Mlc2v-Cre* was not expressed in the outflow tract myocardium although it was found in this tissue by E12.5 (S6 C,D Fig.). Deletion of *Vangl2* in the *Mlc2v-Cre* expression domain resulted in embryos with a normal outflow (n = 6; Fig. 4 K,L,O,P). Thus, although Vangl2 expression is maintained in the outflow myocardium at E12.5 (S6 E,F Fig.), this is not required for outflow tract lengthening and alignment of the the great arteries with the ventricles. Intercrossing of *Vangl2^flox^* mice with *Tie2-Cre* mice, to selectively knock out *Vangl2* in the endocardium, also resulted in mice with normal outflow tract development (S8 Fig.). Thus, these data support the idea that *Vangl2* is required in undifferentiated SHF cells, rather than playing later roles in outflow tract remodelling.

### Vangl2 demarks a transition zone in the distal outflow tract

Having established that *Vangl2* is required in undifferentiated outflow tract precursors (expressing Isl1), derived from the SHF, we next investigated its role in this population. Normally, undifferentiated SHF cells move from the mesothelial dorsal pericardial wall into the distal outflow tract where they begin to differentiate into cardiomyocytes. Thus, we defined a transition zone in the distal outflow tract where SHF-derived cells lose their progenitor phenotype and differentiate (Fig. 5A). We hypothesised that Vangl2 may be playing a key role in this region, regulating this transition from the progenitor to differentiated phenotype. We focussed our studies at E9.0–E9.5, as this is the period just before (E9.0; approximately 17 somite pairs) and as the first abnormalities in the outflow tract wall become apparent (at E9.5; approximately 23 somite pairs).

Vangl2 protein, shown by immunofluorescence, was present in all the cells of the dorsal pericardial and distal outflow tract walls, into the differentiated myocardium, and also in the endocardium (Fig. 5 B–F). However, the spatial distribution of Vangl2 was not uniform throughout this region at E9.0–E9.5. Within the dorsal pericardial and the most distal outflow tract walls, Vangl2 was enriched in the basal cell membrane, as shown by its colocalisation with β-catenin (Fig. 5 B,C,E). Further proximally however, Vangl2 was seen diffusely throughout the cytoplasm (Fig. 5 B,D,F). As mutations that lead to loss of membrane localisation disrupt Vangl2 function and result in the *Lp/Lp* phenotype [33], this suggests that Vangl2 is likely to be playing its major roles in the dorsal pericardial wall or distal outflow tract.

To determine at which stage SHF cells change their expression profile from that of a progenitor to a differentiated cardiomyocyte, relative to the distribution of Vangl2, we analysed the expression of cardiac troponin I, which is expressed in differentiated cardiomyocytes (Fig. 5 G–J). At E9.0, cardiac troponin I immuno-reactivity was found at low levels in the most distal outflow tract wall, but became increasingly abundant proximally as the cells differentiated into cardiomyocytes (Fig. 5I). At the same stage, all cells in the dorsal pericardial wall and in the transition zone expressed Isl1 protein, localised within the nucleus. However, nuclear-localised Isl1 was abruptly lost in more proximal cells, where Vangl2 became cytoplasmic (Fig. 5 K–N). Thus, whereas all three markers overlapped in the most distal outflow tract, loss of membrane-associated Vangl2 corresponded to the loss of nuclear Isl1, defining the proximal boundary of the transition zone where the cells had become differentiated cardiomyocytes.

### Loss of Vangl2 disrupts cellular organisation and polarity in the distal outflow tract

**Figure 6:**
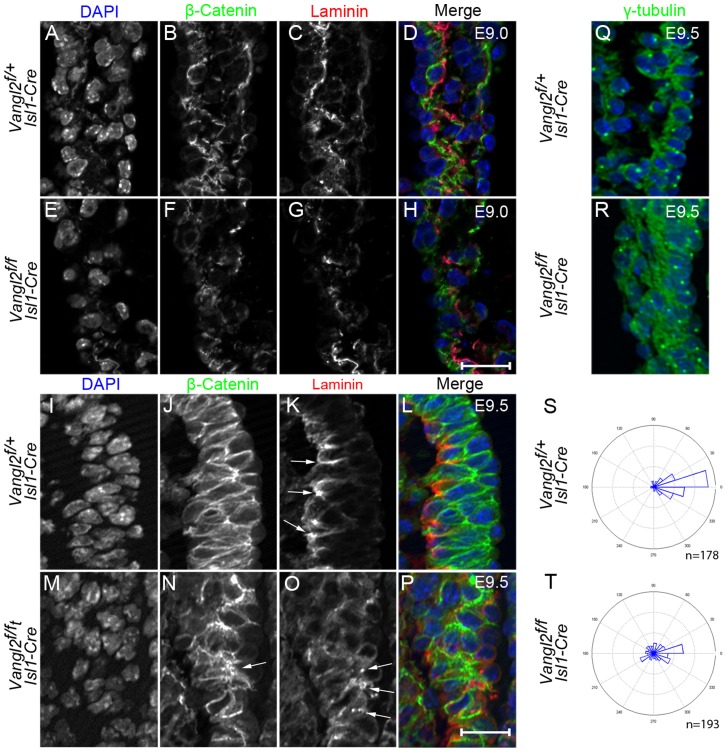
Disruption of epithelial organisation in the distal outflow tract of *Vangl2^flox/flox^; Isl1-Cre* embryos. **A–H**) At E9.0 in control embryos, β-catenin (green; B,D) is localised to the basolateral domain of the cells in the transition zone of the distal outflow wall and laminin (red; C,D) is becoming localised to the basement membrane underlying this. In *Vangl2^flox/flox^; Isl1-Cre* littermates, β-catenin (F,H) and laminin (G,H) are less abundant and the tissue appears disorganised (n = 3). **I–P**) By E9.5, immunofluorescent staining for β-catenin is localised to the basolateral region of cells in the control embryo and shows the pseudo-stratified epithelium of the transition zone (J,L). In contrast, although β-catenin expression is still abundant in the transition zone of *Vangl2^flox/flox^; Isl1-Cre* embryos, the cells appear disorganised and it is difficult to determine its subcellular distribution (N - arrows). Laminin is found basally to the cells of the transition zone in control embryos (K - arrows), but is lost in some places and surrounds other cells within the transition zone of *Vangl2^flox/flox^; Isl1-Cre* embryos (O – arrows, n = 3). Note that whereas the distal outflow wall is 2-3 cell layers thick in the control embryo (L), in some places it is 4-5 cell layers thick in the mutant (P). **Q–T**) γ-tubulin staining of MTOCs at E9.5 shows that these are localised to the apical side of the cells in the distal outflow wall in control embryos (Q and rose plot S). In contrast, the position of the MTOC is much more variable in *Vangl2^flox/flox^; Isl1-Cre* embryos (R and rose plot T), frequently localising to the basolateral side of the cell layer (n = 5) Chi-square, p<0.001. Ap =  Apical, Ba =  Basal, Dis  =  distal, Prox  =  proximal, *Vangl2^f^*  =  *Vangl2^flox^*. Quantification of γ-tubulin performed on 10 embryos (5 control, 5 mutant), with a total of 178 and 193 cells from control and mutant embryos respectively. Scale bar  = 20 µm.

We next wanted to clarify how Vangl2, as a PCP protein that would normally act within the plane of an epithelium, is functioning in the distal outflow tract. We first investigated the distribution of the adherens junction protein β-catenin, which marks the baso-lateral compartment of epithelial cells, together with the extracellular matrix protein laminin, which is found associated with the basal lamina of epithelial cell layers. At E9.0, the distal outflow wall appeared less organised in *Vangl2^flox/flox^; Isl1-Cre* embryos than their control littermates, with reduced staining of β-catenin and laminin (Fig. 6 A–H). This was before outflow shortening was apparent in the mutant embryos. By E9.5, whereas the transition zone had the phenotype of an organised pseudo-stratified epithelium in the controls, the region was markedly disorganised and was becoming thickened in the mutant embryos (Fig. 6 P,R, compare with L,Q, and S5 K,M Fig). β-catenin was lost from the lateral walls of cells of the transition zone in the mutants (Fig. 6N, compare with J), and laminin was absent in some areas of the mutant transition zone and was no longer basally restricted in others (n = 3; Fig. 6P, compare with L). A similar abnormality in the distribution of fibronectin was also observed in the *Vangl2^flox/flox^; Isl1-Cre* embryos (S9 Fig.). Interestingly, laminin was always basally distributed more proximally in the *Vangl2^flox/flox^; Isl1-Cre* outflow tract, where the wall is composed of differentiated cardiomyocytes (S9 Fig.). These findings were highly reproducible. Thus, whereas the cells of the transition zone in the control distal outflow tract had the appearance of a pseudo-stratified epithelium and had polarised expression of epithelial markers, the epithelium was disorganised and thickened in the *Vangl2^flox/flox^; Isl1-Cre* embryos. Moreover, polarised expression of markers was disrupted. In contrast, comparable analyses of *Vangl2^flox/flox^; Nkx2.5-Cre* outflow tract showed only a mild phenotype in the mutant embryos (S9 Fig.), supporting the lack of structural outflow tract abnormalities in this cross.

As asymmetrical cell division is regulated by Vangl2 in some tissues [34] we asked whether disruption of this process might account for the disorganised and thickened epithelium of the transition zone in the mutant embryos. However, we found little or no cell division in the distal outflow tract at E9.0–E9.5, as suggested previously [6], indicating that this is unlikely to be the mechanism underlying the disorganisation observed (S10 Fig.). In order to investigate the possibility of disrupted polarity in more detail, we immuno-stained the microtubule organising centre (MTOC; recognised by γ-tubulin) which is polarised in epithelial cells and localised to the apical side of the cell in the transition zone of control embryos (Fig. 6 Q,S). In contrast, this was much more variable in the transition zone cells of *Vangl2^flox/flox^; Isl1-Cre* embryos (Fig. 6 R,T), with MTOCs commonly found on the basal side of the cell layer. Statistical analysis (Chi Square) showed this difference was highly significant (p<0.001). This suggests that PCP and/or apical-basal polarity is disrupted in the distal outflow wall in the absence of Vangl2.

**Figure 7:**
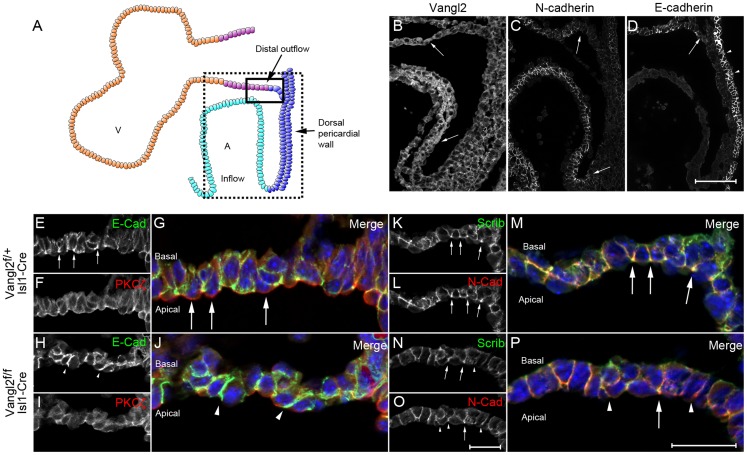
Loss of Vangl2 results in disrupted polarity in the distal outflow tract in *Vangl2^flox/flox^; Isl1-Cre* embryos. **A**) Cartoon representation of a sagittal view of the heart at E9.5, showing both the outflow and inflow regions and the dorsal pericardial wall in between. **B**) Vangl2 is found at the membrane of the distal outflow tract and dorsal pericardial wall, but is cytoplasmic in the myocardium of the heart tube (arrows). **C,D**) E-cadherin and N-cadherin are both found in the distal outflow tract although only N-cadherin is found in the inflow region (C- lower arrow). Neither are expressed at a high level in the dorsal pericardial wall although E-cadherin is expressed strongly in the columnar epithelium of the pharyngeal endoderm (D - arrowheads). **E–J**) Within the transition zone of the distal outflow tract, E-cadherin is enriched apically (E,G – see arrows in E); this enrichment is lost in the mutant embryos (H,J, n = 3). PKCζ is apically restricted in control embryos (F,G – see arrows in G). This apical restriction is lost in the *Vangl2^flox/flox^; Isl1-Cre* embryos (I,J, n = 3). **K–P**) Similar to E-cadherin, Scrib and N-cadherin are apically enriched in control embryos (K-M - arrows). This enrichment is generally lost in the cells from the mutant embryos (N-P - arrowheads), although some can still be observed (P – arrow, n = 3) *Vangl2^f^*  =  *Vangl2^flox^*. Scale bar  = 100 µm (B-D), 50 µm (E**–**P).

We studied the phenotype of the SHF-derived cells more closely as they moved from the mesothelial dorsal pericardial wall into the outflow tract, by looking at other markers associated with polarised epithelia that are expressed in an apical-basal polsarised manner. E-cadherin, an adherens junction-associated protein of epithelial cells was expressed only at low levels in the dorsal pericardial wall, while a related junctional protein, N-cadherin, was not detected in this tissue (Fig. 7 A–D), supporting the idea that this is not a typical epithelium. Within the pseudo-stratified epithelium of the distal outflow tract however, both of these proteins were expressed in a polarised manner, being enriched at the apical-basal boundary (Fig. 7 E,G,L,M). This suggests that as cells move into the distal outflow tract they take on a more overt epithelial phenotype. As in control embryos, E-cadherin expression was up-regulated in the distal outflow of *Vangl2^flox/flox^; Isl1-Cre* mutants at E9.0–E9.5, in comparison to the dorsal pericardial wall. However, it was mislocalised with staining found throughout the baso-lateral compartment of the cells (Fig. 7 E,G,H,J and S11 Fig.). The subcellular localisation of N-cadherin expression was also perturbed in the distal outflow tract of *Vangl2^flox/flox^; Isl1-Cre* embryos, with loss of enrichment at the apical-basal boundary (Fig. 7 L,M,O,P).

We analysed other proteins known to be compartmentally restricted in epithelial cells. Analysis of PKCζ in the distal outflow tract revealed that whilst the protein was markedly enriched in the apical compartment of cells in the transition zone of control embryos, this apical enrichment was lost in *Vangl2^flox/flox^; Isl1-Cre* embryos (Fig. 7 F,G,I,J and S11 Fig.). Scrib has been implicated in regulating epithelial cell adhesion and has been shown to physically interact with Vangl2 in epithelial cells [35], although it does not do so in the early myocardium [36]. Scrib was localised to the baso-lateral compartment of cells within the distal outflow tract wall in control embryos, but was enriched at the apical-basal boundary. In *Vangl2^flox/flox^; Isl1-Cre* embryos, Scrib was no-longer enriched at the apical-basal boundary (arrows in Fig. 7 K,M,N,P). This abnormality was restricted to the distal outflow tract, with normal Scrib expression seen in the dorsal pericardial wall and pharyngeal endoderm although both of these tissues also express *Isl1-Cre* (S11 Fig.), and in epidermis, which does not express *Isl1-Cre*. Thus, deletion of Vangl2 from SHF cells results in the disruption of both PCP and apical-basal polarisation and loss of epithelial phenotype in the distal outflow tract wall.

### Vangl2 retains SHF cells as undifferentiated progenitors

**Figure 8:**
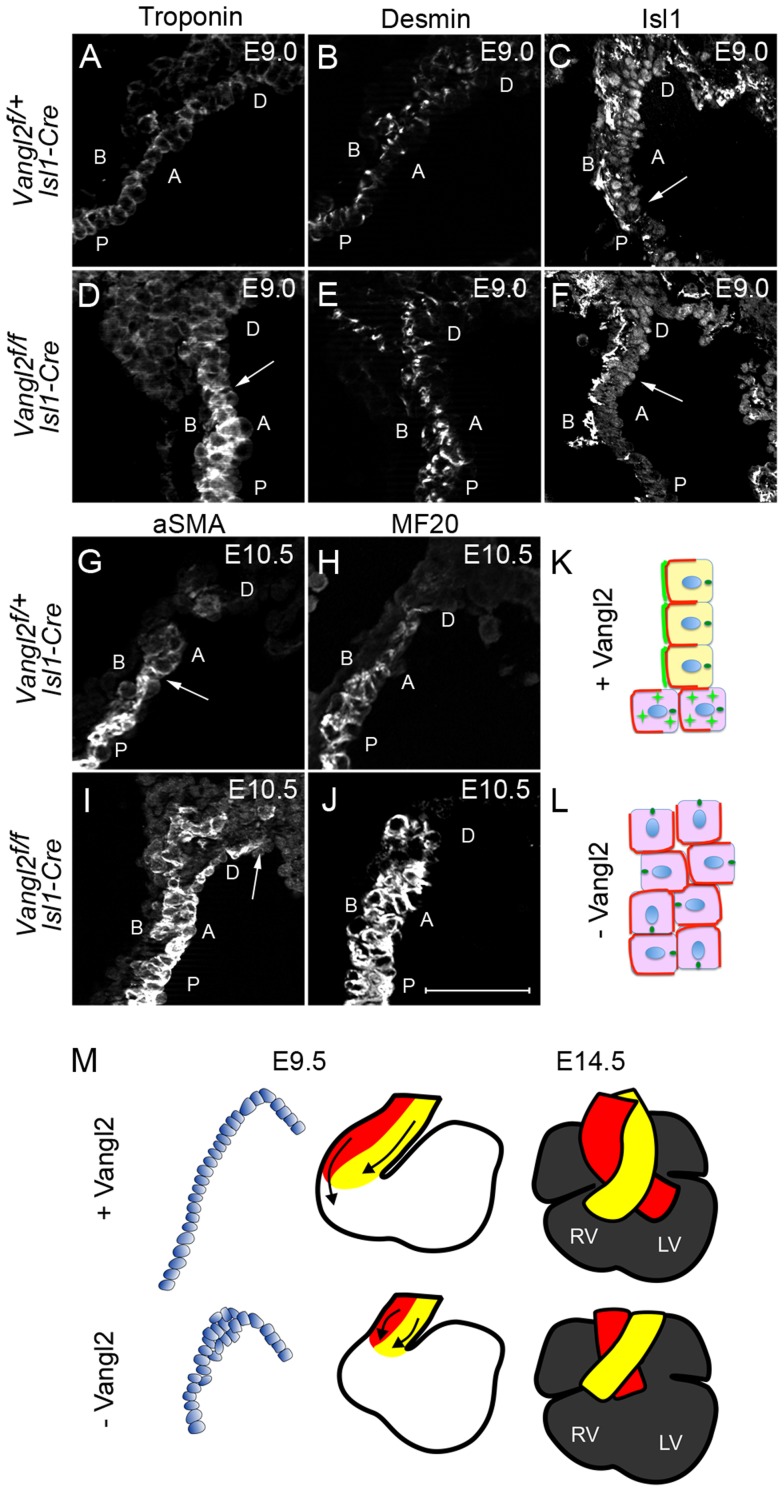
Loss of Vangl2 results in loss of SHF progenitor phenotype and premature differentiation in the distal outflow tract of *Vangl2^flox/flox^; Isl1-Cre* embryos. **A–F**) At E9.0, cardiac troponin I expression is low distally and increases proximally through the outflow tract of control embryos (A). In contrast, high-level expression is found more distally in *Vangl2^flox/flox^; Isl1-Cre* embryos (D, n = 2). Desmin, which is expressed at high level in cardiomyocytes and at lower level by smooth muscle cells (B) is also increased within the distal outflow tract of *Vangl2^flox/flox^; Isl1-Cre* embryos (E, n = 3). Whereas Isl1 is localised to the nucleus of control embryos throughout an extended region of the distal outflow tract, defining the transition zone (C - arrows), it is significantly reduced in the nuclei of cells in the distal outflow of *Vangl2^flox/flox^; Isl1-Cre* embryos (F – arrows point to the proximal extent of the staining, n = 3). G–J) Similar to E9.0, at E10.5, both αSMA (G,I, n = 3) and MF20 (H,J; staining myosin heavy chain, n = 3) are expressed more distally in the outflow tract of *Vangl2^flox/flox^; Isl1-Cre* embryos than in stage-matched littermates. K,L) Cartoon showing distribution of Vangl2 (bright green) at the boundary of the transition zone in the distal outflow tract of control embryos, where it is localised to the membrane through the transition zone, but is cytoplasmic (green stars) more proximally. Basolateral markers are represented in red and the MTOC, localising to the apical side of the cell, in dark green (K). In the absence of Vangl2, basolateral marker domains are expanded and the MTOC, although still apically positioned, is rotated in many cells. The wall is also thickened (L). M) Model showing how loss of epithelial phenotype of the cells within the distal outflow tract wall at E9.5 could result in a shortened outflow tract and double outlet right ventricle by E14.5. A =  Apical, B =  Basal, D =  distal, P =  proximal, *Vangl2^f^*  =  *Vangl2^flox^*. Scale bar  = 100 µm.

We lastly wanted to establish how the disruption of polarity and loss of epithelial phenotype impacted on the fate of the cells as they move through the distal outflow walls. We investigated whether the expression of either cardiomyocyte differentiation markers or Isl1 was altered in the distal outflow wall of *Vangl2^flox/flox^; Isl1-Cre* mutants at E9.0–E10.5. At E9.0 we observed that cardiac troponin I was expressed initially at very low levels within the distal outflow wall of control embryos, becoming progressively more strongly expressed proximally (as shown in Fig. 5). In contrast, high-level expression appeared more distally in *Vangl2^flox/flox^; Isl1-Cre* littermates (Fig. 8 A,D). Desmin, which is expressed at elevated levels by cardiomyocytes, but is also found at lower levels in smooth muscle cells at this stage, was also expressed at higher levels more distally in the mutant embryos (Fig. 8 B,E). As expected, we observed a similar disorganisation of Isl1-expressing cells in the distal outflow of *Vangl2^flox/flox^; Isl1-Cre* as was observed in *Lp/Lp* embryos (Fig. 8 C,F). However, it was striking that nuclear Isl1 localisation was lost more distally in the distal outflow wall of *Vangl2^flox/flox^; Isl1-Cre* than in control embryos. Notably there was a significant amount of non-nuclear Isl1 observed in cells in control and mutant outflow walls (Fig. 8 C,F). To confirm the findings of premature differentiation in the distal outflow tract of *Vangl2^flox/flox^; Isl1-Cre*, we examined the expression of other differentiation markers at E10.5. Similar results to those observed for cardiac troponin I were found using an antibody to myosin heavy chain (MF20; Fig. 8 H,J). Moreover, when we examined the expression of αSMA, which at these stages labels both smooth muscle cells and differentiating cardiomyocytes, it was also expressed at high levels more distally in the mutant embryos than in control littermates (Fig. 8 G,I). Thus, *Vangl2^flox/flox^; Isl1-Cre* cells lose their progenitor phenotype and differentiate earlier than those of control embryos. Together, our data suggest that Vangl2 is required to establish the epithelial phenotype of cells in the distal outflow tract wall and that this epithelialisation is required to maintain SHF-derived cells in an organised, polarised state which maximises outflow tract lengthening. This allows normal alignment of the great arteries with the ventricular chambers

## Discussion

The spontaneously occurring *Lp* mutant has been used to study the role of *Vangl2* in cardiac development [26], [27], [30]. However, the gross morphological defects that are characteristic of *Lp/Lp*, including craniorachischisis and incomplete axial rotation, limit its use in dissecting out the role of *Vangl2* during heart morphogenesis. We originally suggested that the cardiac looping defects observed in the *Lp/Lp* may be secondary to the severe neural tube closure and axial turning defects [26]. Indeed, the aortic arch abnormalities that are highly penetrant in *Lp/Lp* and in the *Vangl2^flox/flox^; Sox2-Cre* and *Vangl2^flox/flox^; PGK-Cre* mutants described here, may be secondary to these gross abnormalities in body form, as they are only observed in the presence of craniorachischisis. In contrast, we show that the outflow tract anomalies are primary defects. The complexity added by the presence of other embryonic defects, together with the contributions of a number of different cell populations to the developing outflow tract, makes analysis of a global loss of Vangl2 inadequate for the purpose of establishing its precise role in heart development. Here we have described the generation of a conditionally targeted deletion of *Vangl2*, which when expressed globally recapitulates the outflow anomalies observed in *Lp/Lp*. Using this model, we have shown that the expression of *Vangl2* in the *Isl1-Cre* expressing SHF is essential for the normal development of the outflow tract.

Our data suggests that it is the expression of *Vangl2* in the undifferentiated SHF (*Isl1-Cre* expressing) population of the distal outflow tract that is critical for outflow tract development, as its deletion in the *Mlc2v-Cre*-expressing outflow myocardium and *Tie2-Cre*-expressing endothelium led to normal outflow tract development. However, as well as finding outflow tract shortening as early as E9.5, abnormalities in movement of muscle cells from the outflow tract wall into the cushions (the process of myocardialisation) was also found in the *Vangl2^flox/flox^; Isl1-Cre* embryos at E13.5, as in *Lp/Lp* embryos [30], showing that the outflow myocardium does not recover from its early abnormalities. In the distal outflow tract wall, cells transition from an undifferentiated form (expressing nuclear Isl1 protein) to differentiated myocardium (with a consequent loss of nuclear Isl1 and upregulation of striated muscle markers including myosin heavy chain and cardiac troponin I). Intriguingly, Vangl2 is lost from the cell membrane and is localised to the cytosol at the proximal boundary of the transition zone, where the cells differentiate to myocardium. Thus, this switch from membrane to cytoplasmic Vangl2, together with the loss of nuclear Isl1, defines the proximal boundary of the transition zone. There is good evidence to suggest that Vangl2 is active when it is membrane-associated, as mutations that block membrane-association block function [33]. Thus, we suggest that it is the membrane-localisation of Vangl2 in the transition zone that imparts planar polarity on the cells, maintaining their epithelial phenotype. When this membrane-association is lost, the cells lose their typical epithelial appearance and differentiate. Although *Vangl1*, a close homologue of *Vangl2* is expressed in the early embryo, its expression domain is more restricted than that of *Vangl2* [37],[38]. *Vangl1* mutants do not have an outflow tract phenotype [37]. Thus, it seems likely that *Vangl2* is the principal *Vangl* gene acting in the early heart. We did not see an outflow tract phenotype when we inter-crossed the *Vangl2^flox^* mice with *Nkx2.5-Cre* line. However, analysis of the *Nkx2.5-Cre* expression domain showed that although it was expressed early enough to delete Vangl2 in cardiomyocyte progenitors [31], its expression was patchy in the distal outflow in our hands, and Vangl2 expression was maintained in these cells. Thus, these data are consistent with the idea that Vangl2 is required in undifferentiated cells, rather than differentiated derivatives of the SHF.

During outflow tract elongation in chickens, cells move from a proliferative pool within the dorsal pericardial wall into the distal outflow not as individual cells, but as a cohesive sheet [6]. This is similar to the movements of epithelial sheets in other organ systems [39], [40]. Although this mechanism of addition of cells to the outflow has not been confirmed in mammals, it seems likely that an analogous process takes place. Once they reach the region of the distal outflow, our data suggests the SHF-derived cells then acquire a more epithelial phenotype (i.e. robust polarised expression of E-cadherin) than they exhibited while they were in the mesothelium of the dorsal pericardial wall. Within this newly epithelialized tissue, Vangl2 signalling regulates the apical-basal polarised expression of a range of markers of stratified epithelial tissues, including Scrib and PKCζ [41], [42]. We see markedly abnormal distribution of these markers in the distal outflow tract wall of *Vangl2^flox/flox^; Isl1-Cre* embryos, with a reduced apical domain and expanded basolateral domain. Moreover, analysis of the position of MTOCs in the cells of the distal outflow tract suggests that planar polarisation of cells is also disturbed within this tissue. Thus, both apical-basal and planar cell polarity appear to be regulated by Vangl2 within the early outflow tract (Fig. 8 K,L).

We propose that the polarisation of the cells within the distal outflow tract and the consequent acquisition of epithelial phenotype is linked to the elongation of the outflow vessel. Specifically, we propose that the organisation of the cells into a pseudo-stratified epithelium ensures that the outflow tract wall lengthens rather than thickens. Epithelia are characterised by cells with strong lateral attachments (including E-cadherin-containing adherens junctions) that keep the cells in a sheet. Our data suggests that Vangl2 plays a key role in regulating planar polarity in the distal outflow wall, such that the cells are similarly oriented within the plane of the epithelium. When Vangl2 is lost from these cells in the distal outflow wall, cells are rotated relative to their neighbours, resulting in mis-positioning of the junctional complexes and disruption of the characteristic epithelial phenotype of the tissue (see Fig. 8 K-M for simplified cartoon). Our observation that Vangl2 also appears to be regulating the apical-basal positioning of markers, may add to this phenotype by further disrupting the positioning of cell junctions. Thus, Vangl2 appears to be essential for forming/maintaining the sheet-like structure of the cells that is necessary for epithelial tube formation. The thickened, shortened outflow tract seen in the absence of Vangl2 in some ways resembles the consequences of disrupted PCP signalling at gastrulation in vertebrate embryos, when convergence and extension movements narrow and lengthen the developing embryo (reviewed in [18]). Both processes likely involve junctional remodelling [40], however, the similarities in final-phenotype may be misleading as the SHF cells are added to the distal end of the lengthening outflow tract, whereas convergence and extension gastrulation movements occur intrinsically within a fixed pool of cells. Indeed, the process we describe does not seen to be directly analogous to any other mammalian organ system so far described. However, there are similarities with the described roles for PCP genes in tracheal tube lengthening in *Drosophila* [43] and *Vang1* (the only worm *vang/strabismus* gene) specifically in *C.elegans* intestinal tube formation [44]. In both these cases, disruption of PCP signalling results in disorganisation of polarised markers and cell-cell relationships, and a shortened epithelial tube. During the early development of the outflow tract, undifferentiated SHF cells, expressing Isl1, move from the mesothelium of the dorsal pericardial wall into the outflow tract before differentiating to cardiomyocytes. This process happens prematurely in the *Vangl2^flox/flox^; Is1-Cre* mutants. Thus, the acquisition of the organised epithelial phenotype may be important both physically to create length within the tubular structure of the distal outflow, but also to prevent premature differentiation of the cells. Whether the latter contributes directly to the phenotype remains unclear. Interestingly, *Wnt5a*, an activator of PCP signalling, has been shown to be regulated by *Tbx1* [45], one of the key transcription factors involved in maintaining SHF cells in a proliferative, undifferentiated state [10]. Thus, Vangl2 may be acting with these other factors in a network that regulates the addition of SHF cells to the lengthening outflow tract.

The link between a shortened outflow tract and double outlet right ventricle is well recognised [46], [47]. The on-going, regulated addition of cells from the SHF is crucial for the elongation of the vessel, a process that is required to position the proximal regions of the vessels so that they can align appropriately with the ventricular chambers. In the absence of Vangl2, the outflow tract thickens rather than lengthens. This shortened, thicker outflow tract is unable to align correctly with the ventricular chambers, resulting in double outlet right ventricle and ventricular septal defects (Fig. 8M). Whether this mechanism could explain cardiac alignment defects such as double outlet right ventricle in humans is the subject of on-going studies.

Disruption of *Vangl2*, either throughout the body or just in the SHF lineage, results in double outlet right ventricle and ventricular septal defects. Notably, we saw no abnormalities when *Vangl2* was deleted in NCC, despite PCP signalling having been shown to play crucial roles in NCC migration in frogs and zebrafish [17]. Indeed, a recent study has shown that Vangl signalling is dispensable for NCC migration in mice [38], suggesting that NCC migration is regulated differently in mammals than in frogs, fish and birds. Similar outflow malformations to those we observe when *Vangl2* is deleted specifically in the SHF lineage are observed in mice carrying mutations in other PCP genes including *Dvl 1-3, Wnt5a*, *Wnt11* and *Fz1/Fz2* [48], [49], [50], [51], [52]. Whilst there are likely to be multiple causes of double outlet right ventricle, the strong relationship between this abnormality and mutations in PCP pathway genes suggests that PCP signalling may be fundamental to the normal septation and alignment of the great arteries with the ventricular chambers. As well as ultimately developing double outlet right ventricle, *Wnt11^-/-^* mutants display abnormalities at earlier stages of heart development. These include a reduction in outflow tract length and perturbation in the cytoarchitecture of outflow tract cardiomyocytes [51]. TGFβ2 signalling was shown to be acting downstream of Wnt11 in the outflow myocardium, and *Wnt11* null and *Tgfβ2* null embryos showed abnormalities in apical-basal markers in the outflow wall at E11.5 [51]. Although earlier stages were not analysed, and thus a direct comparison with our study cannot be made, it is possible that TGFβ2, acting downstream of Wnt11 and Vangl2, might be involved in maintaining organisation of the distal outflow tract wall and thus regulate outflow tract lengthening. More recently, Sinha et al, [53] studied the abnormalities in outflow tract morphogenesis in *Dvl2* mutants. They concluded that the defects resulted from abnormalities in the incorporation of SHF progenitors from the splanchnic mesoderm into the dorsal pericardial wall, prior to movement into the outflow vessel. Vangl2 is expressed in the dorsal pericardial wall, however despite close examination, we saw no abnormalities in this area. Sinha et al (2012; [53]) did not describe the phenotype of the cells within the distal outflow tract in the *Dvl2* mutants, or examine markers of polarised cells in their embryos. Thus it is unclear whether the mechanism we describe could also be a component of the *Dvl2* mutant phenotype. However, *Wnt11* is expression is restricted to the outflow tract myocardium [51] suggesting that this might be a key factor in activating PCP and thereby Vangl2 signalling in the distal outflow tract. Thus, it seems likely that PCP signalling, via Vangl2 and Wnt11 (and/or Wnt5a), is playing an essential role in elongating the distal outflow tract, facilitating on-going cardiac morphogenesis.

## Methods

### Ethics statement

All animals were maintained and killed according to the requirements of the Animals (Scientific Procedures) Act 1986 of the UK Government. This work was approved by the Newcastle University Ethical Review Committee and conformed to Directive 2010/63/EU of the European Parliament.

### Mouse strains and embryos

*Loop-tail* mice from the *LPT/Le* inbred strain were originally obtained from Professor Andrew Copp (UCL, London). *Vangl2^floxneo^* mice were created in partnership with Ozgene (Australia). The mice were subsequently crossed with *FlpE* [54] mice to generate *Vangl2^flox^* mice and then inter-crossed with *ROSA-Stop-eYFP* [55] mice to allow Cre-based lineage tracing. Cre driver lines, including *Sox2-Cre* [56], *PGK-Cre* [57], *Isl1-Cre* [58], *Wnt1-Cre* [59], *Mlc2v-Cre* [60], and *Nkx2.5-Cre* [30] were all intercrossed with the *Vangl2^flox^* line and *ROSA-Stop-eYFP* mice. All mice were maintained on the C57Bl/6 background (Charles River) and were backcrossed for a minimum of three generations. For all experiments, transgenic mice were compared with their wild type and heterozygote littermates. Mice were bred and embryos collected according to standard protocols [32].

### Genotyping of *Vangl2^flox^* mice

*Vangl2^flox^* mice were genotyped using genomic DNA isolated from ear clips or limb buds using primers: forward: CCGCTGGCTTTCCTGCTGCTG; reverse: TCCTCGCCATCCCACCCTCG.

### *In situ* hybridization

Embryos were dissected and fixed in 4% paraformaldehyde (PFA) in DEPC-PBS (phosphate buffered saline) overnight. The following day, the embryos were washed in PBS, dehydrated sequentially in 25%, 50% and 75% methanol in PBT (0.1% Tween 20 in PBS) on ice, and then stored at −20°C in 100% methanol until use. When required, embryos were rehydrated through the reverse methanol series as above and then equilibrated in PBT. The embryos were bleached in 6% hydrogen peroxide in PBT for 1 hour to inactivate endogenous peroxidase in the embryos, and washed three times in PBT. To improve the penetration of the probe into the embryo, they were treated with proteinase K (PK, 5 µg/ml) at RT for 7 minutes (in case of E10.5 embryos). Glycine (2 mg/ml) was added to stop the PK activity and the embryos were gently rocked for 5 minutes. After two washes in PBT, the embryos were refixed in 0.2% glutaraldehyde in 4% PFA, and then rocked for 20 minutes. 1 ml of prehybridization solution was added to the embryos in a 2 ml tube and they were incubated at 70°C for 2 hours. After discarding the prehybridization solution, 500 µl of hybridisation solution including the DIG-labeled RNA probe (1 µg/ml) was replaced and then incubated at 70°C overnight. The next day, the embryos were washed twice in salt solution I and II (Solution I, 50% formamide, 5x SSC, pH 4.5, 1% SDS; Solution II, 50% formamide, 2x SSC, pH 4.5) at 70°C for 30 minutes, respectively. Embryos were washed three times for 5 minutes at RT temperature in freshly made MABT and non-specific antigens blocked by incubating in a 10% blocking solution (Roche) in MABT for 1 hour. Anti-DIG antibody (Roche) was added to 1% blocking solution/MABT at a concentration of 1∶5000 and was left to pre-absorb at 4°C for 1 hour. 500 µl of the antibody solution was added to each embryo and they were left at 4°C for two nights with gentle rocking to allow complete penetration of the antibody. After this incubation, embryos were washed three times for 5 minutes in TBST then 5 times of 1 hour washes in TBST at RT. Embryos were then given 3 times of 10 minutes washes in NTMT to prepare them for development. Embryos were transferred to glass bottles and 3ml of NBT/BCIP (1, 4-nitro blue tetrazolium chloride/5-bromo-4-chloro-3-indoyl-phosphate) added to each at a concentration of 18 µl/ml of NTMT. Embryos were left to develop in the dark until the desired level of staining was achieved. Once the reaction was completed, the embryos were washed in PBT twice for 5 minutes, and stored in the dark in PBT containing 0.48 µg/ml of thymerisol to prevent fungal growth.

### RT-PCR

Total RNA extraction from embryonic tissue was carried out using 1 ml Trizol-Reagent (Ambion) per sample, according to the manufacturer's instructions. Samples were quantified via spectrophotometry, and cDNA generated, using 1 µg of total RNA as a template, with superscript III reverse transcriptase (Life technologies). PCR was carried out using the following primers: TGAGGGCCTCTTCATCTCC, ACCAATAACTCCACGGG.

### Wax embedding and haematoxylin and eosin staining

Following dissection at the appropriate stage of development, embryos were washed in PBS, and then fixed by immersion in 4% paraformaldehyde in PBS at 4°C for 1 to 3 nights dependent on their age. Embryos were subsequently dehydrated in ethanol and processed for wax sectioning. Sections were cut at 8 µm using a rotary microtome (Leica). For haemotoxylin and eosin staining, slides were de-waxed with two 10 minute washes of Histoclear and were hydrated to water through an ethanol/H_2_0 gradient (100%, 90% 70% and 50%). Slides were placed in Ehrlich haematoxylin (RA Lamb) for 10 minutes then were transferred into a trough of running tap water. The slides were left until the sections changed colour from purple to blue. The sections were differentiated by dipping in acid alcohol (1% HCl in 70% ethanol) for 10–30 seconds, and then were placed back into tap water until the blue colour was restored. When an acceptable intensity of haemotoxylin stain was achieved, the slides were transferred into 1% aqueous eosin for 5 minutes, rinsed in tap water, then dehydrated through the same ethanol gradient, before washing twice in Histoclear and mounting in Histomount (National Diagnostics).

### Production of the Vangl2 antibody

The Vangl2 antibody, produced by C. Dean, was raised in rabbit against the following Vangl2 specific peptide: CLAKKVSGFKVYSLGEENST by 21^st^ Century Biochemicals, MA, USA and validated by western blot on lysate from HEK293 cells transfected with a Vangl2-GFP construct. A band representing the GFP-tagged construct was detected just above the 75KDa marker using either the Vangl2 antibody or an anti-GFP antibody.

### Immunohistochemistry

Slides were de-waxed with Histoclear and rehydrated through a series of ethanol washes. Following washes in PBS, antigen retrieval was performed by boiling slides in citrate buffer (0.01 mol/L) for 10 minutes. Samples were blocked in 10% FCS and then incubated either overnight at 4°C, or at room temperature for 2 hours with the following antibodies: E-cadherin, β-catenin, N-Cadherin (BD Transduction Laboratories), fibronectin, Scrib, PKCζ (Santa Cruz), Isl1, MF20 (Developmental studies Hybridoma Bank, University of Iowa), GFP, alpha smooth muscle actin (Abcam), gamma tubulin, laminin (Sigma), cardiac troponin I (HyTest), desmin (Millipore). For immunofluorescence, samples were incubated at room temperature for two hours, with secondary antibodies conjugated to either Alexa 488 or Alexa 594 (Life Technologies). Fluorescent slides were washed then mounted with Vectashield Mounting medium with DAPI (Vector Labs). For non fluorescent staining, samples were incubated with biotinylated secondary antibodies for 1 hour, then with AB complex (Vector labs) for a further hour. Slides stained with DAB were washed then counter-stained with 5% methyl green. After dehydration in 100% butanol and Histoclear, slides were mounted using Histomount.

### Quantification of cell polarity

Cells were stained with the γ–tubulin antibody to identify the microtubule organising centre (MTOC). The orientation of the cell was defined by the angle between the most apical extent of the cell, the centre of the cell and the MTOC, and was measured using the angle tool in ImageJ. MTOCs lying proximally relative to the apex were considered to have an obtuse angle, and were transformed as such. Angles were converted to radians and plotted using the rose plot function in MATLAB. To analyse the distribution of MTOCs in control and mutant outflow tracts, eight sectors of possible MTOC cell position were defined (0–44°, 45–89°… 315–359°) and the distribution of MTOCs within these sectors compared by Chi-Square (IBM, SPSS statistics, version 21).

### β-gal staining

Cells with an active *lacZ* gene in embryos carrying both the *Wnt1-Cre* and *ROSA26R* constructs stain blue when treated with X-Gal. Embryos were washed twice in PBS and fixed in a solution containing 0.1 M phosphate buffer, 2% PFA, 5 mM EGTA (pH 8.0), 0.2% glutaraldehyde and 2 mM MgCl_2_. They were washed twice in wash buffer (0.1 M phosphate buffer, 0.01% Na-deoxycholate, 0.02% Nonidet-P40, 2 mM MgCl_2_) and X-Gal stained at 37°C wrapped in aluminium foil overnight. X-Gal solution contains 10 mM K-ferrocyanide, 10 mM K-ferricyanide and 1 mg/ml X-Gal in wash buffer. Stock X-Gal powder is dissolved in dimethylformamide before adding to the staining solution. The next day, after rinsing in PBS, the embryos were fixed in 4% PFA and embedded in wax as described above.

### Western blotting

Tissue samples from embryos were lysed with 500 µl 1X laemmli buffer (2% SDS, 5% betamercaptoethanol), 10% glycerol, 0.05% w/v bromophenol blue, 0.0625M Tris-HCl pH 6.8) and run on pre-cast 10% poly-acrylamide gels (Biorad). Samples were transferred to PVDF (ImmobilonP Millipore) in ice cold transfer buffer (48 Mm Tris-HCl pH 8, 39 mM glycine, 0.04% SDS, 20% methanol) for 1 hour at 4°C. Membranes were washed in TBST (2.4% w/v Trizma hydrochloride, 8% w/v sodium chloride at pH 7.6; 0.1% Tween-20) then blocked in 5% milk/TBST at room temperature for 1 hour. The membrane was incubated with primary antibody (Vangl2 1∶2500/GAPDH 1∶25000 in 5% milk/TBST) overnight at 4°C, washed then incubated with secondary antibody (1∶2500, Dako) for 1 hour at room temperature. Membranes were washed then developed with ECL substrate (SuperSignal West Dura, Thermo Scientific) on Amersham Hyperfilm ECL (GE healthcare).

### Graphics

Images were manipulated in Photoshop CS3 (Adobe). Diagrams were created using CorelDRAW X5 (Corel).

## Supporting Information

S1 Fig Expression of chamber markers in *Lp/Lp* embryos at E10.5. **A,B**) *Nppa*, **C,D**) *Mlc2a* and **E,F**) *Mlc2v* expression in *Lp/+* and *Lp/Lp* embryos showing normal expression patterns but abnormal heart loop in *Lp/Lp*. In each case the right ventricle is hypoplastic and/or the outflow tract is shortened in the *Lp/Lp* embryos compared to controls.
(TIF)

S2 Fig External and cardiac defects in *Vangl2^floxneo^*, *Vangl2^flox^; Lp; Sox2-Cre* and *Vangl2^flox^; Lp; PGK-Cre* at E14.5. Prior to crossing with *FlpE* mice, the *Vangl2^flox^* targeting vector retained the *neomycin* selection cassette. To establish its affect on Vangl2 expression, *Vangl2^floxneo/+^* mice were inter-crossed to generate *Vangl2^floxneo/floxneo^* mice. In each case, the arrows point to the neural tube defects. **A**) *Vangl2^floxneo/+^* embryos exhibit a normal external phenotype. **B**) Transverse sectioning revealed no abnormalities in either the heart or the pharyngeal arch arteries. **C,E,G**) *Vangl2^floxneo^* mutants show variability in their external phenotype. Of the three *Vangl2^floxneo/floxneo^* embryos examined, two exhibited craniorachischisis and had the looped tail observed in *Lp/Lp* mice (C,E), while one displayed spina bifida only (G). **D,F,H**) Transverse sections revealed that all *Vangl2^floxneo/floxneo^* mice exhibited heart defects including retro-oesophageal right subclavian artery (RORSA), where the aorta forms a ring around oesophagus (D), DORV (F) and VSD (H). **I-P**) *Vangl2^flox^; Lp; Sox2-Cre* (K,L) and *Vangl2^flox^; Lp; PGK-Cre* (O,P) mice exhibit the same external phenotype as *Lp/Lp* and *Vangl2^flox/flox^; Sox2Cre* mice, exhibiting craniorachischisis and a looped tail, as observed in *Lp/Lp* mice. Transverse sections reveal heart malformations as observed in *Lp/Lp* mice including RORSA, double outlet right ventricle and ventricular septal defect. DORV - double outlet right ventricle, RSA - right subclavian artery, VSD - ventricular septal defect, Scale bar  = 2 mm (white), 500 µm (black).
(TIF)

S3 Fig External and cardiac defects in *Vangl2^flox/flox^; Sox2-Cre* embryos and *Vangl2^flox/flox^; PGK-Cre* at E14.5. *Vangl2^flox/flox^; Sox2-Cre* and *Vangl2^flox/flox^; PGK-Cre* embryos display an abnormal external phenotype and cardiac defects. **A–B**) Additional cardiovascular defects observed in *Vangl2^flox/flox^; Sox2-Cre mice*, including ventricular septal defect and double outlet right ventricle. **C**) *Vangl2^flox/+^; PGK-Cre* embryos display a normal external phenotype. **D**) A transverse section through the heart reveals no cardiac defects, with the normal outlet of the aorta from the left ventricle. **E**) *Vangl2^flox/flox^; PGK-Cre* embryos exhibited craniorachischisis and a looped tail as observed in *Lp/Lp* mice. **G–H**) Transverse sections of the hearts of *Vangl2^flox/flox^; PGK-Cre* embryos reveal a number of defects including double outlet right ventricle (F,G; in G both the aorta and pulmonary trunk can be seen exiting the right ventricle) and ventricular septal defect (H). DORV - double outlet right ventricle, VSD - ventricular septal defect. Scale bar  = 2 mm (white), 500 µm (black).
(TIF)

S4 Fig **Vangl2 is not expressed by NCC and its expression remains unaltered in** *Vangl2^flox/flox^; Wnt1-Cre* **embryos at E10.5.** **A–D**) NCC (green) are abundant in the pharyngeal region and distal outflow tract of control embryos. However, close examination (B′,C′) shows that Vangl2 (red) does not localise to the NCC. **E–H**) Comparable areas of *Vangl2^flox/flox^; Wnt1-Cre* embryos shows that the expression pattern of both Vangl2 and the distribution of NCC is comparable to control embryos. High power images (F′,G′ show lack of localisation of Vangl2 to NCC).
(TIF)

S5 Fig *Vangl2^flox/flox^; Isl1-Cre* embryos have a shortened outflow tract at E9.5 and display various cardiac defects by E14.5. **A–I**) *Vangl2* can be efficiently deleted from the SHF lineage using *Isl1-Cre*. While Vangl2 protein (red in merged images) is abundant in the outflow tract of *Vangl2^flox/+^; Isl1-Cre* embryos (C), the protein is not detectable in *Vangl2^flox/flox^; Isl1-Cre* embryos within the *Isl1-Cre* expression domain (green in merged images D,H,I; labelled by eYFP) of the developing outflow tract (H). Vangl2 protein is retained outside of the *Isl1-Cre* expression domain, for example in the neural tube and otic vesicles (arrows in I). **J–M**) At E9.5 Isl1-positive cells can be seen moving into the distal outflow, which results in lengthening of the outflow tract. *Vangl2^flox/flox^; Isl1-Cre* embryos display a significantly shorter outflow tract than stage-matched controls (compare arrows in J and L) even at this early stage of development. Isl1-positive cells within the distal outflow of *Vangl2^flox/flox^; Isl1-Cre* embryos appear disorganised and the tissue of the distal outflow tract is malformed. **N–S**) At E14.5 a number of cardiac defects can be seen within *Vangl2^flox/flox^; Isl1-Cre* embryos including VSD (N,Q) and DORV (O,R). No arch artery defects can be seen in *Vangl2^flox/flox^; Isl1-Cre* however (P,S). Scale bar  = 500 µm.
(TIF)

S6 Fig A,B Myocardialisation is abnormal in *Vangl2^flox/flox^; Isl1-Cre* embryos at E13.5. Cardiac troponin I staining shows that cardiomyocytes extend into the outflow cushions in control embryos at E13.5 (A). This is much reduced in *Vangl2^flox/flox^; Isl1-Cre* embryos. **C,D**) *Mlc2v-Cre* expression at E10.5-E12.5. *Mlc2v-Cre* (blue) is not found in the outflow myocardium at E10.5 although it is apparent in the proximal outflow tract myocardium by E12.5 (arrows), before myocardialisation begins. **E,F**) Vangl2 is localised to the cytoplasm of cardiomyocytes in the outflow tract at E12.5. Vangl2 is maintained in the outflow tract myocardium at E12.5, with localisation throughout the cytoplasm. Arrows point to cells in the outflow wall.
(TIF)

S7 Fig *Nkx2.5-Cre* lineage tracing as shown by *Cre*-activated GFP at E10.5. **A–C**) *Nkx2.5-Cre* expression as indicated by *Cre*-based lineage labelling can be seen within the dorsal pericardial wall and the distal outflow tract (A). The expression of GFP within this tissue is patchy however (A,B) suggesting that *Nkx2.5-Cre* levels are low within this region, resulting in the *Cre* being unable to efficiently drive GFP expression. Vangl2 expression in retained in the distal outflow tract of *Vangl2^flox/flox^; Nkx2.5-Cre* embryos at E9.5 (C).
(TIF)

S8 Fig No outflow phenotype in *Vangl2^flox/flox^; Tie2-Cre* embryos. The outflow tract is septated (A) and the aorta exits from the left ventricle (B) in *Vangl2^flox/flox^; Tie2-Cre* embryos at E15.5.
(TIF)

S9 Fig Loss of Vangl2 affects tissue organisation in the distal outflow tract. **A–H** β-catenin and laminin are only subtly disrupted in the distal outflow tract of *Vangl2^flox/flox^; Nkx2.5-Cre* embryos at E9.5, supporting the evidence that some Vangl2 is retained in the distal outflow wall of these embryos. **I–N**) Fibronectin is normally laid down as a constituent part of the basal lamina and so is basally restricted in the distal outflow tract walls of control embryos (D-F). In *Vangl2^flox/flox^; Isl1-Cre* mutants, however, fibronectin can be seen surrounding cells throughout the outflow walls (L-N). **O–T**) Unlike the distal outflow tract where laminin distribution is abnormal in the *Vangl2^flox/flox^; Isl1-Cre* mutants, laminin is basally restricted in the proximal outflow tract in both controls (O-Q) and mutants (R-T). **U**) Diagrammatic representation of the outflow tract E9.5. Sections shown in panels A-N were taken from the distal outflow (upper box) whereas sections in panels O-T were from the proximal outflow (lower box). Scale bar  = 20 µm
(TIF)

S10 Fig Absence of proliferation in cells in the distal outflow tract wall at E9.5. **A,B**) There is little or no proliferation in cells in the distal outflow tract wall (arrows in B) at E9.5. **A,C**) However, extensive proliferation is seen in the nearby pharyngeal arch (arrows in C).
(TIF)

S11 Fig Disruption of epithelial organisation in the distal outflow tract of *Vangl2^flox/flox^; Isl1-Cre* embryos at E9.0. **A–H**) Similarly to at E9.5, E-cadherin and aPKCζ are mislocalised in the distal outflow tract of mutant embryos (E–H), compared with stage-matched littermates (A–D). Notably, aPKCζ is found basally (arrow in H) rather than apically in some cells in the mutant embryo. **I–T**) Scrib staining was normal in *Vangl2^flox/flox^; Isl1-Cre* mutants in the epidermis which does not express *Isl1-Cre* (O-Q, compare to I-K) and in the dorsal pericardial wall which is *Isl1-Cre*-positive (compare R–T with L–N).
(TIF)
