# Supplementary figures and images for: Vangl2-Regulated Polarisation of Second Heart Field-Derived Cells Is Required for Outflow Tract Lengthening during Cardiac Development
Source: PLoS Genet. 2014 Dec 18;10(12):e1004871. doi: 10.1371/journal.pgen.1004871 (PMC4270488; doi:10.1371/journal.pgen.1004871)

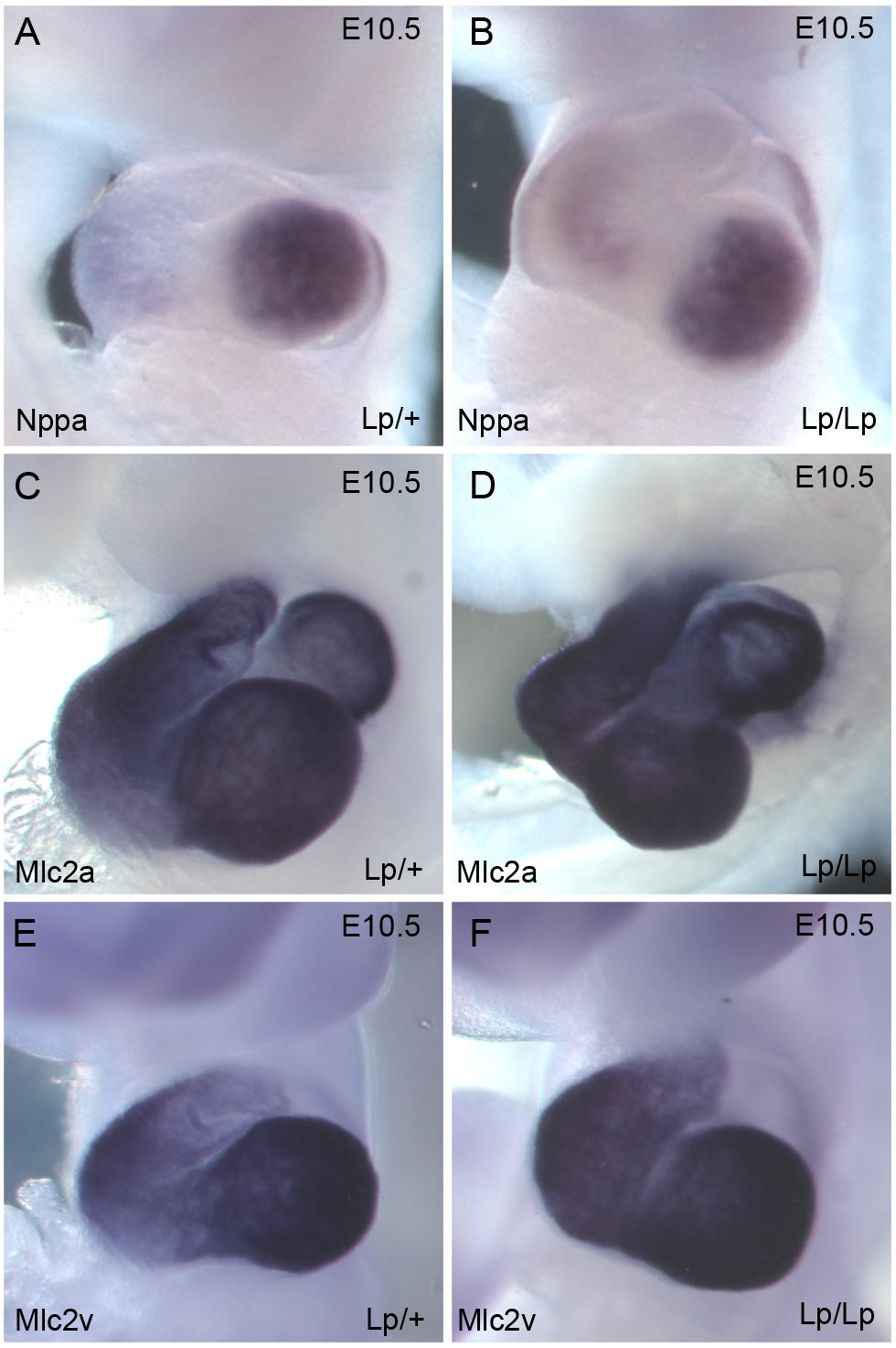

Supplement: S1 Fig — Expression of chamber markers in Lp/Lp embryos at E10.5. A,B) Nppa, C,D) Mlc2a and E,F) Mlc2v expression in Lp/+ and Lp/Lp embryos showing normal expression patterns but abnormal heart loop in Lp/Lp. In each case the right ventricle is hypoplastic and/or the outflow tract is shortened in the Lp/Lp embryos compared to controls. (TIF) [file pgen.1004871.s001.tif]

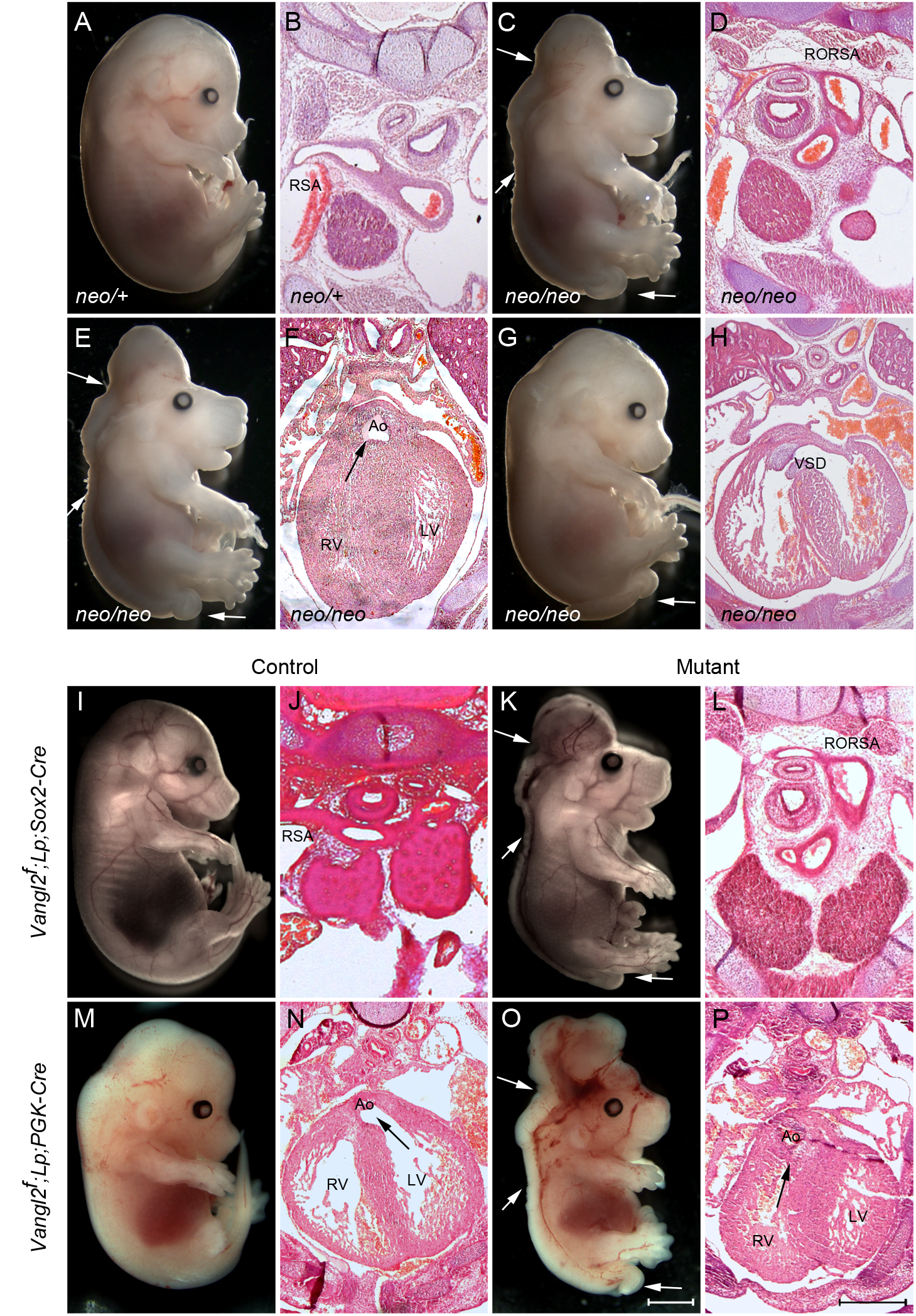

Supplement: S2 Fig — External and cardiac defects in Vangl2floxneo, Vangl2flox; Lp; Sox2-Cre and Vangl2flox; Lp; PGK-Cre at E14.5. Prior to crossing with FlpE mice, the Vangl2flox targeting vector retained the neomycin selection cassette. To establish its affect on Vangl2 expression, Vangl2floxneo/+ mice were inter-crossed to generate Vangl2floxneo/floxneo mice. In each case, the arrows point to the neural tube defects. A) Vangl2floxneo/+ embryos exhibit a normal external phenotype. B) Transverse sectioning revealed no abnormalities in either the heart or the pharyngeal arch arteries. C,E,G) Vangl2floxneo mutants show variability in their external phenotype. Of the three Vangl2floxneo/floxneo embryos examined, two exhibited craniorachischisis and had the looped tail observed in Lp/Lp mice (C,E), while one displayed spina bifida only (G). D,F,H) Transverse sections revealed that all Vangl2floxneo/floxneo mice exhibited heart defects including retro-oesophageal right subclavian artery (RORSA), where the aorta forms a ring around oesophagus (D), DORV (F) and VSD (H). I-P) Vangl2flox; Lp; Sox2-Cre (K,L) and Vangl2flox; Lp; PGK-Cre (O,P) mice exhibit the same external phenotype as Lp/Lp and Vangl2flox/flox; Sox2Cre mice, exhibiting craniorachischisis and a looped tail, as observed in Lp/Lp mice. Transverse sections reveal heart malformations as observed in Lp/Lp mice including RORSA, double outlet right ventricle and ventricular septal defect. DORV - double outlet right ventricle, RSA - right subclavian artery, VSD - ventricular septal defect, Scale bar = 2 mm (white), 500 µm (black). (TIF) [file pgen.1004871.s002.tif]

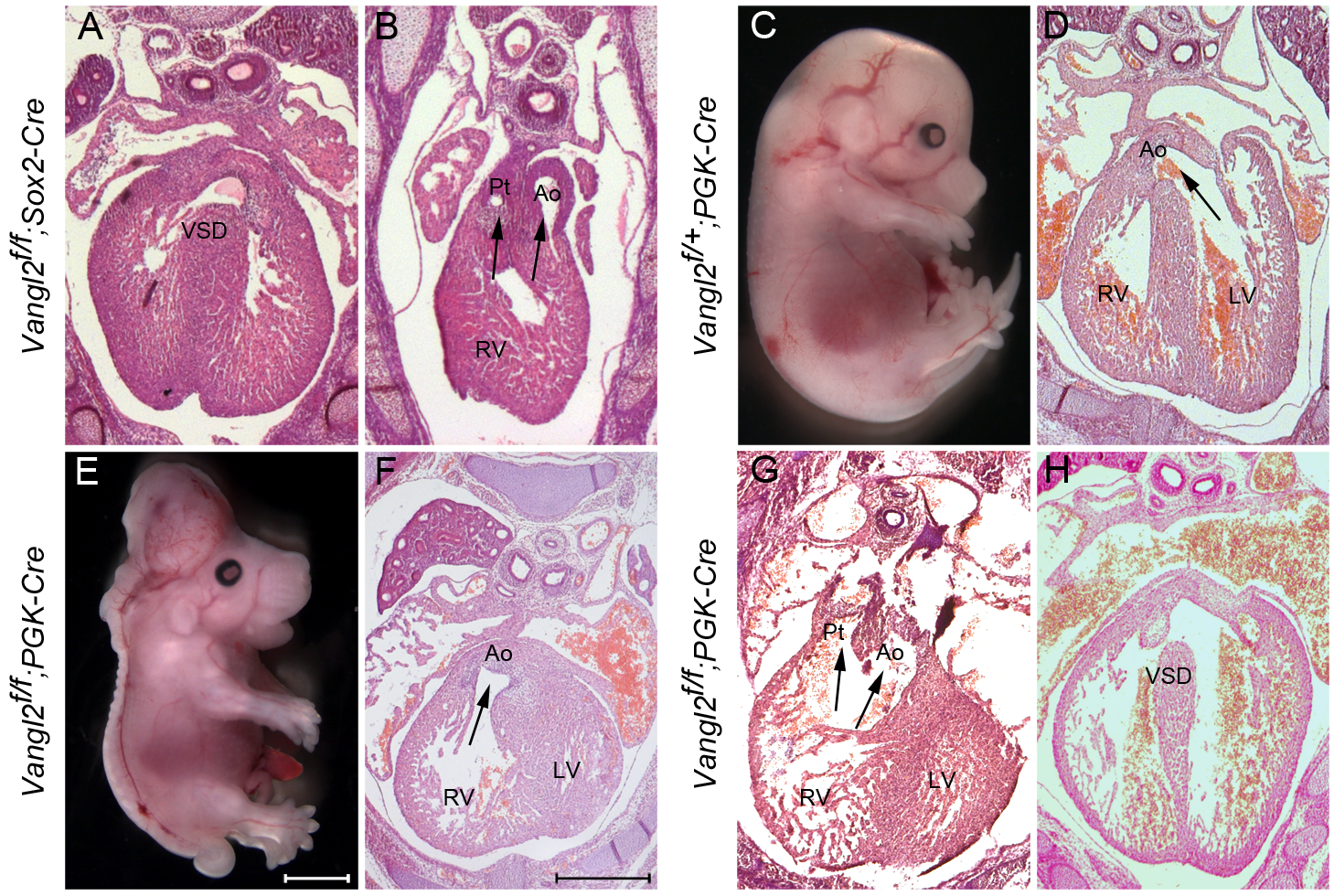

Supplement: S3 Fig — External and cardiac defects in Vangl2flox/flox; Sox2-Cre embryos and Vangl2flox/flox; PGK-Cre at E14.5. Vangl2flox/flox; Sox2-Cre and Vangl2flox/flox; PGK-Cre embryos display an abnormal external phenotype and cardiac defects. A–B) Additional cardiovascular defects observed in Vangl2flox/flox; Sox2-Cre mice, including ventricular septal defect and double outlet right ventricle. C) Vangl2flox/+; PGK-Cre embryos display a normal external phenotype. D) A transverse section through the heart reveals no cardiac defects, with the normal outlet of the aorta from the left ventricle. E) Vangl2flox/flox; PGK-Cre embryos exhibited craniorachischisis and a looped tail as observed in Lp/Lp mice. G–H) Transverse sections of the hearts of Vangl2flox/flox; PGK-Cre embryos reveal a number of defects including double outlet right ventricle (F,G; in G both the aorta and pulmonary trunk can be seen exiting the right ventricle) and ventricular septal defect (H). DORV - double outlet right ventricle, VSD - ventricular septal defect. Scale bar = 2 mm (white), 500 µm (black). (TIF) [file pgen.1004871.s003.tif]

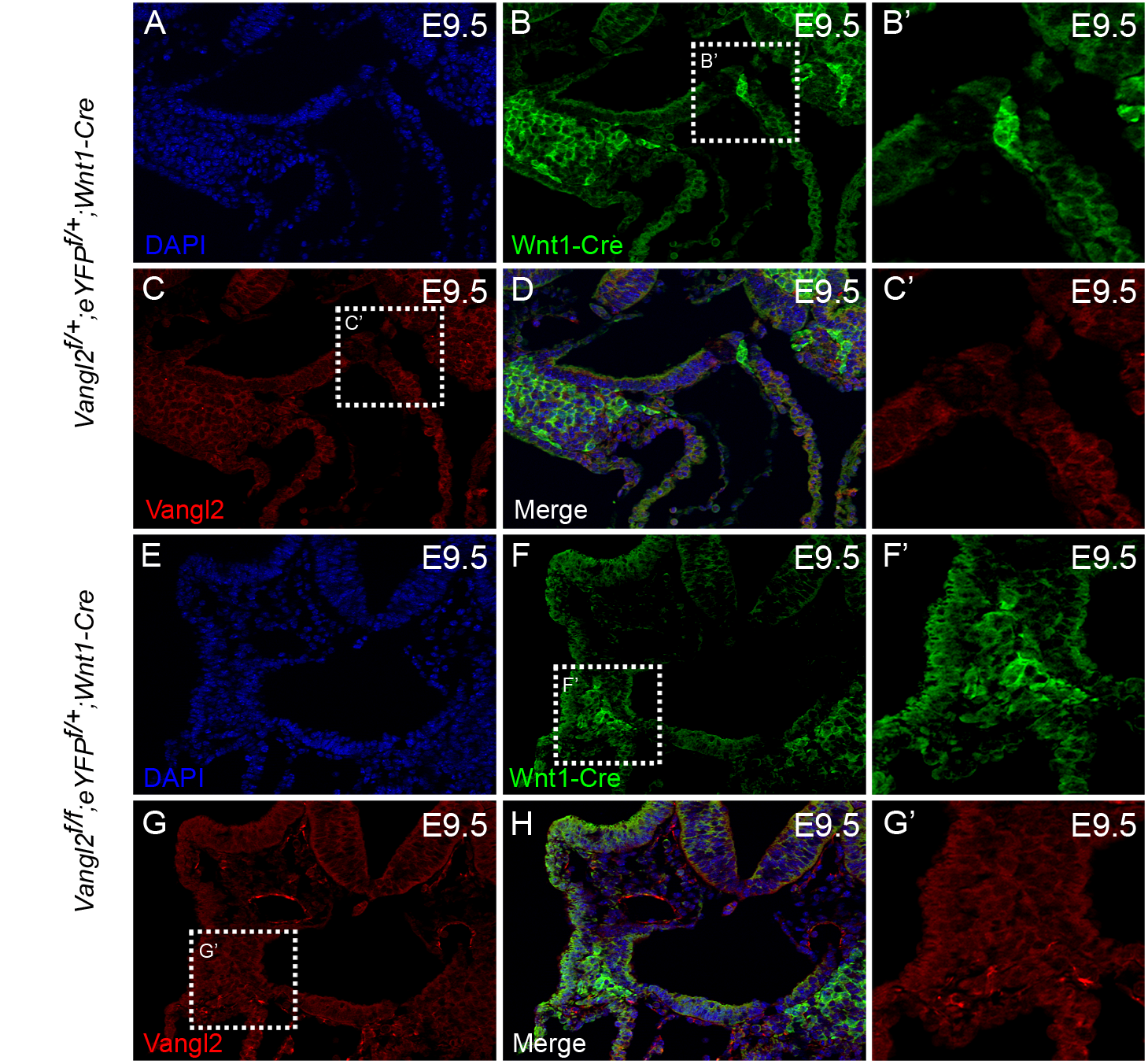

Supplement: S4 Fig — Vangl2 is not expressed by NCC and its expression remains unaltered in Vangl2flox/flox; Wnt1-Cre embryos at E10.5. A–D) NCC (green) are abundant in the pharyngeal region and distal outflow tract of control embryos. However, close examination (B′,C′) shows that Vangl2 (red) does not localise to the NCC. E–H) Comparable areas of Vangl2flox/flox; Wnt1-Cre embryos shows that the expression pattern of both Vangl2 and the distribution of NCC is comparable to control embryos. High power images (F′,G′ show lack of localisation of Vangl2 to NCC). (TIF) [file pgen.1004871.s004.tif]

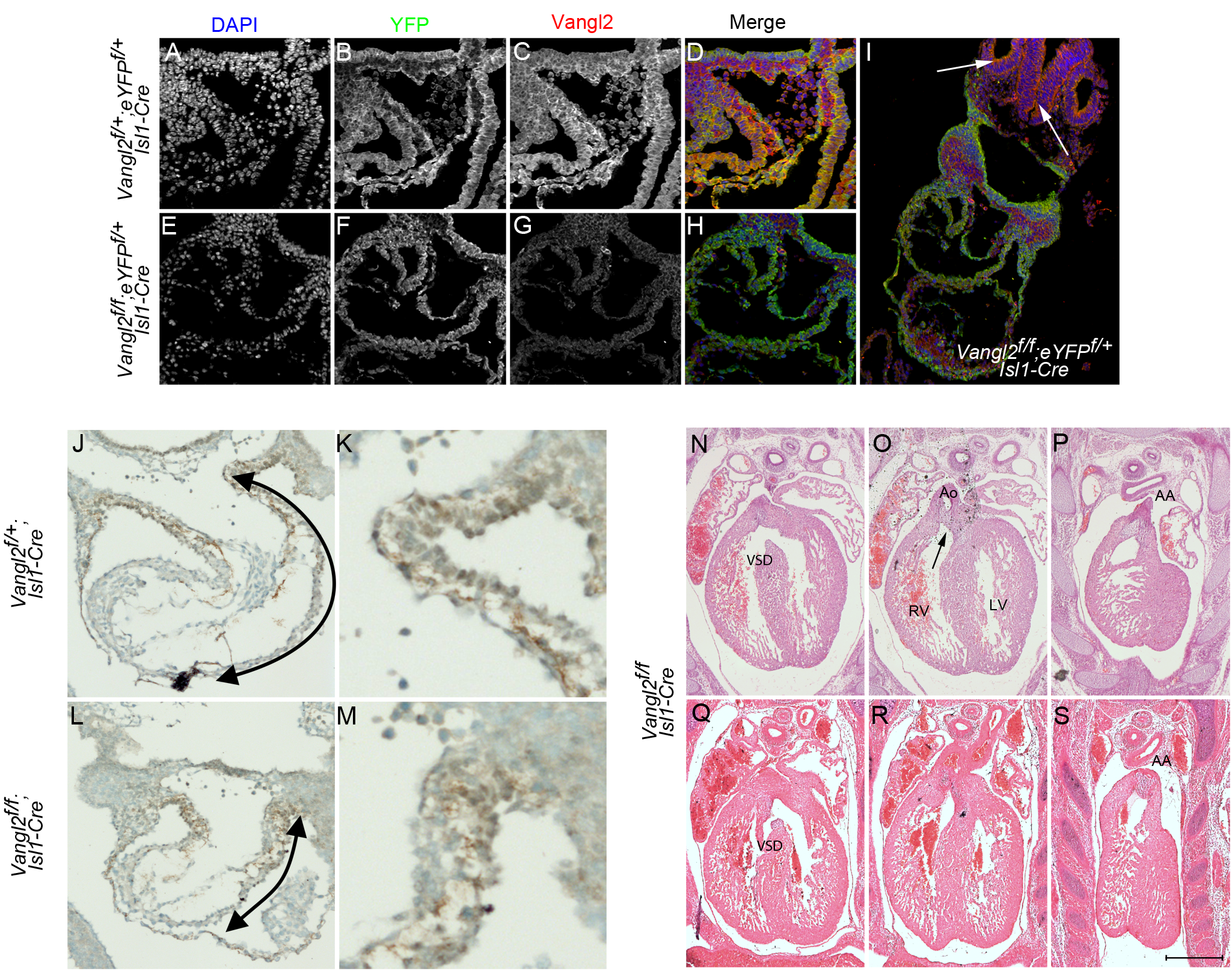

Supplement: S5 Fig — Vangl2flox/flox; Isl1-Cre embryos have a shortened outflow tract at E9.5 and display various cardiac defects by E14.5. A–I) Vangl2 can be efficiently deleted from the SHF lineage using Isl1-Cre. While Vangl2 protein (red in merged images) is abundant in the outflow tract of Vangl2flox/+; Isl1-Cre embryos (C), the protein is not detectable in Vangl2flox/flox; Isl1-Cre embryos within the Isl1-Cre expression domain (green in merged images D,H,I; labelled by eYFP) of the developing outflow tract (H). Vangl2 protein is retained outside of the Isl1-Cre expression domain, for example in the neural tube and otic vesicles (arrows in I). J–M) At E9.5 Isl1-positive cells can be seen moving into the distal outflow, which results in lengthening of the outflow tract. Vangl2flox/flox; Isl1-Cre embryos display a significantly shorter outflow tract than stage-matched controls (compare arrows in J and L) even at this early stage of development. Isl1-positive cells within the distal outflow of Vangl2flox/flox; Isl1-Cre embryos appear disorganised and the tissue of the distal outflow tract is malformed. N–S) At E14.5 a number of cardiac defects can be seen within Vangl2flox/flox; Isl1-Cre embryos including VSD (N,Q) and DORV (O,R). No arch artery defects can be seen in Vangl2flox/flox; Isl1-Cre however (P,S). Scale bar = 500 µm. (TIF) [file pgen.1004871.s005.tif]

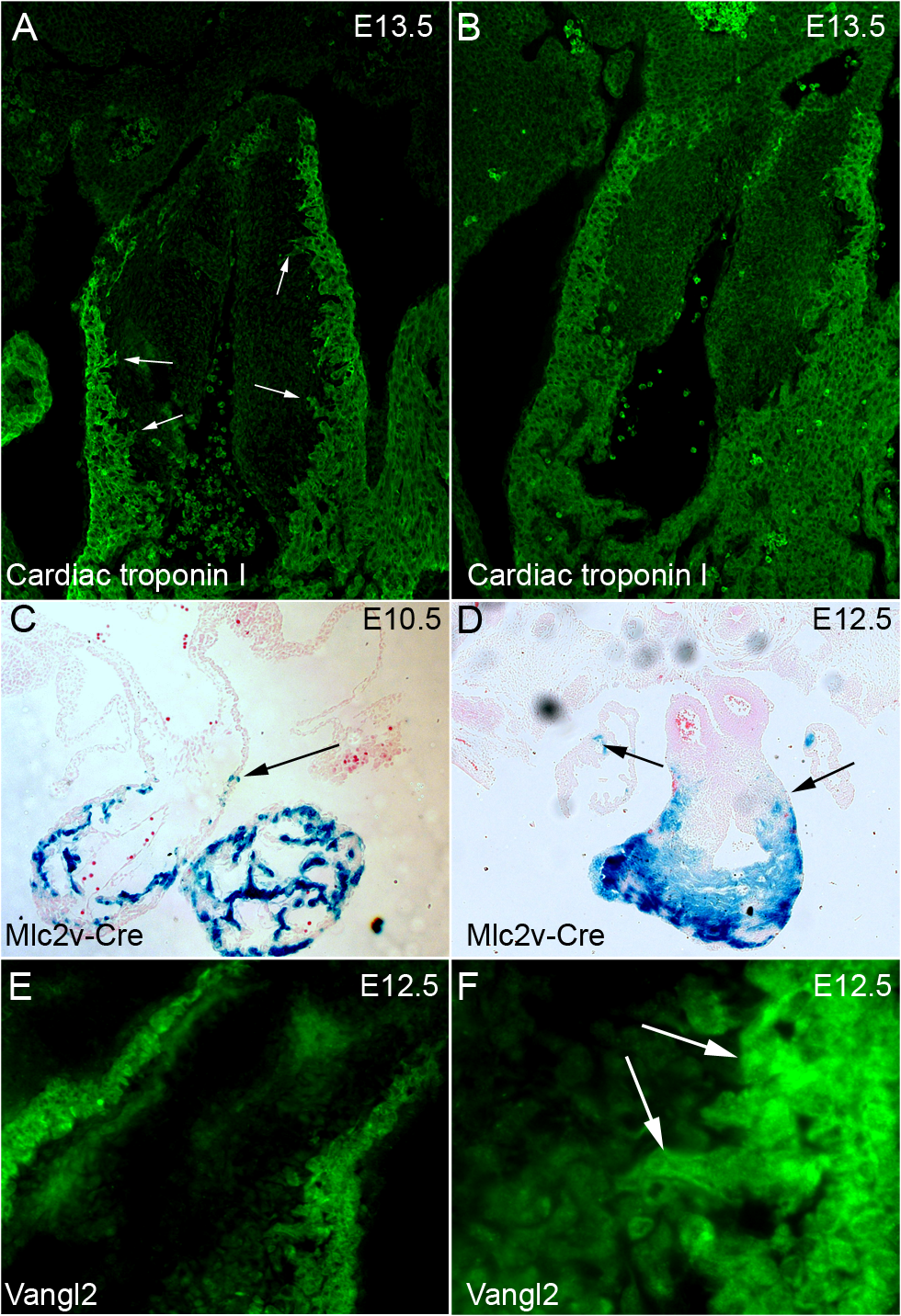

Supplement: S6 Fig — A,B Myocardialisation is abnormal in Vangl2flox/flox; Isl1-Cre embryos at E13.5. Cardiac troponin I staining shows that cardiomyocytes extend into the outflow cushions in control embryos at E13.5 (A). This is much reduced in Vangl2flox/flox; Isl1-Cre embryos. C,D) Mlc2v-Cre expression at E10.5-E12.5. Mlc2v-Cre (blue) is not found in the outflow myocardium at E10.5 although it is apparent in the proximal outflow tract myocardium by E12.5 (arrows), before myocardialisation begins. E,F) Vangl2 is localised to the cytoplasm of cardiomyocytes in the outflow tract at E12.5. Vangl2 is maintained in the outflow tract myocardium at E12.5, with localisation throughout the cytoplasm. Arrows point to cells in the outflow wall. (TIF) [file pgen.1004871.s006.tif]

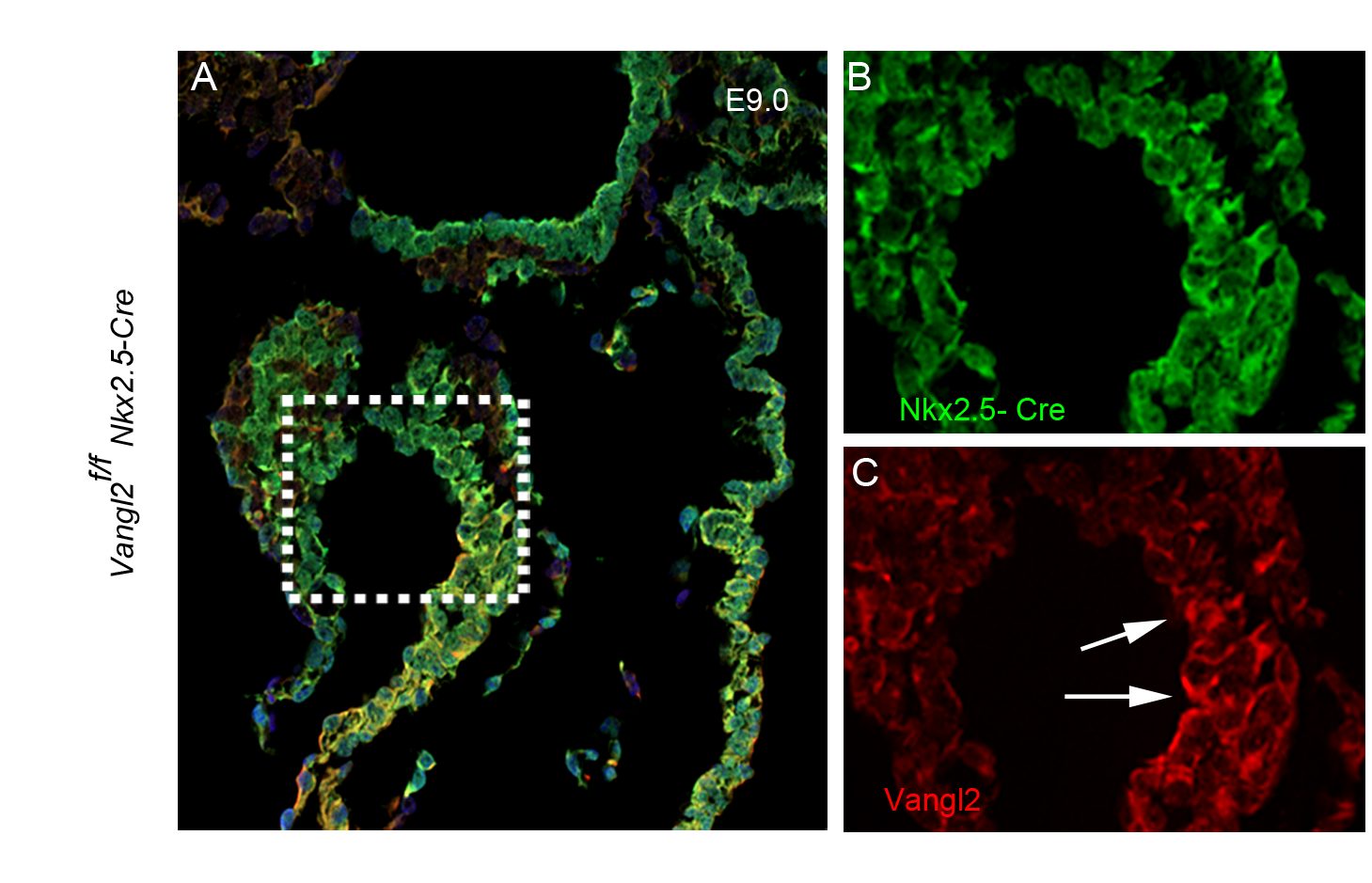

Supplement: S7 Fig — Nkx2.5-Cre lineage tracing as shown by Cre-activated GFP at E10.5. A–C) Nkx2.5-Cre expression as indicated by Cre-based lineage labelling can be seen within the dorsal pericardial wall and the distal outflow tract (A). The expression of GFP within this tissue is patchy however (A,B) suggesting that Nkx2.5-Cre levels are low within this region, resulting in the Cre being unable to efficiently drive GFP expression. Vangl2 expression in retained in the distal outflow tract of Vangl2flox/flox; Nkx2.5-Cre embryos at E9.5 (C). (TIF) [file pgen.1004871.s007.tif]

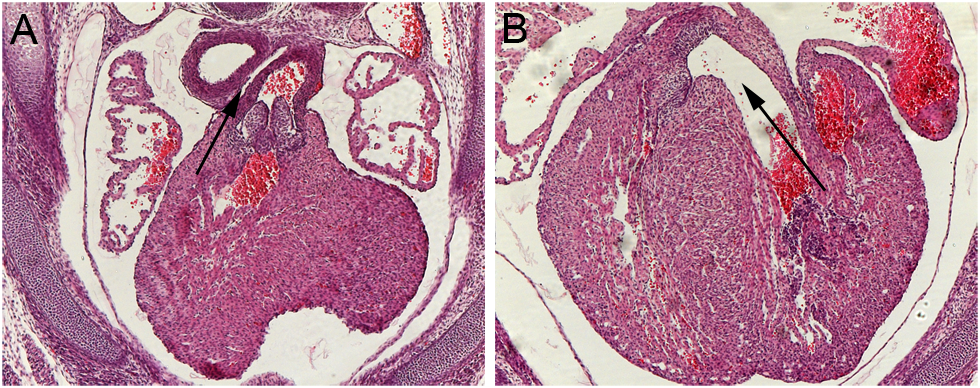

Supplement: S8 Fig — No outflow phenotype in Vangl2flox/flox; Tie2-Cre embryos. The outflow tract is septated (A) and the aorta exits from the left ventricle (B) in Vangl2flox/flox; Tie2-Cre embryos at E15.5. (TIF) [file pgen.1004871.s008.tif]

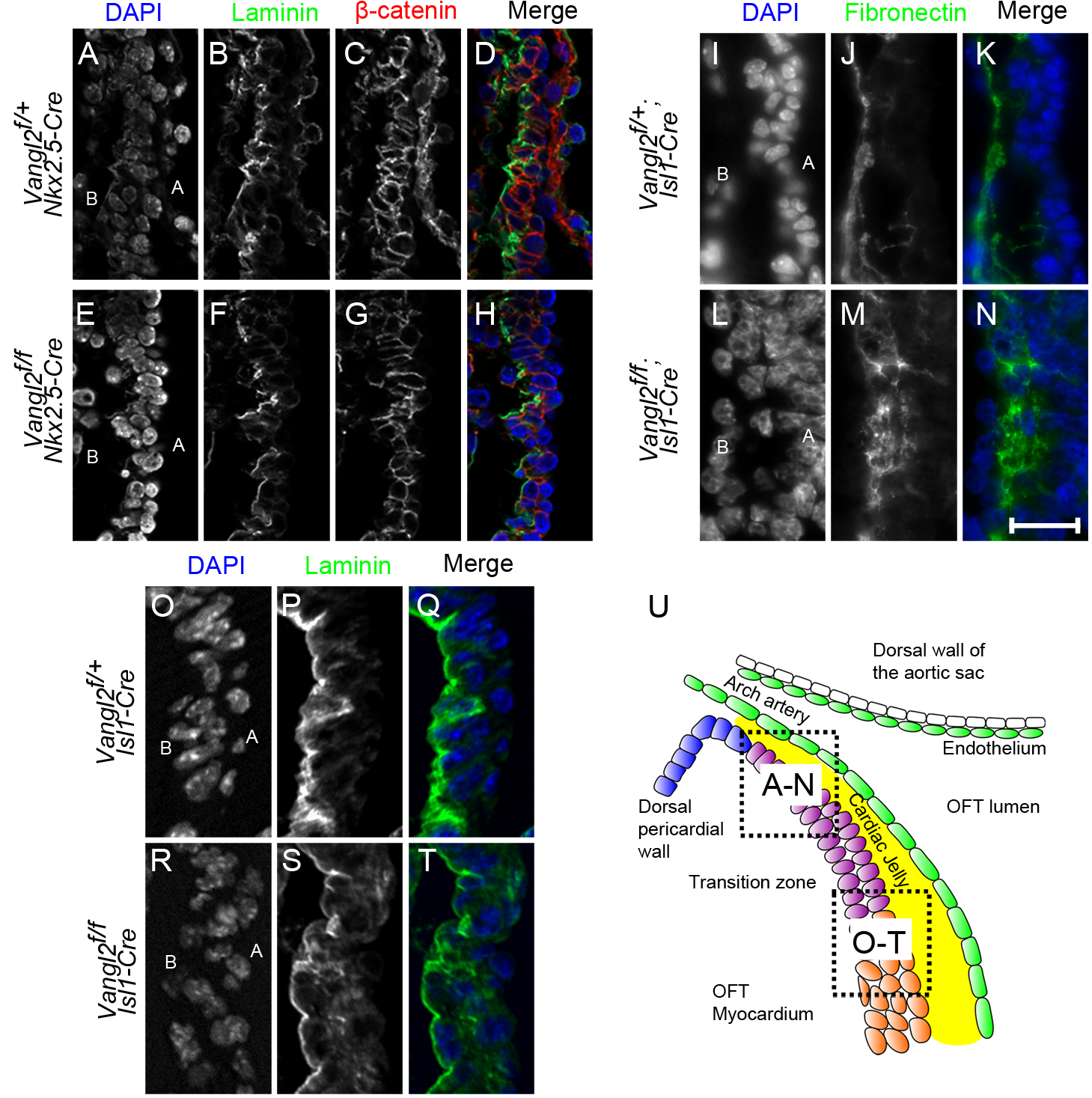

Supplement: S9 Fig — Loss of Vangl2 affects tissue organisation in the distal outflow tract. A–H β-catenin and laminin are only subtly disrupted in the distal outflow tract of Vangl2flox/flox; Nkx2.5-Cre embryos at E9.5, supporting the evidence that some Vangl2 is retained in the distal outflow wall of these embryos. I–N) Fibronectin is normally laid down as a constituent part of the basal lamina and so is basally restricted in the distal outflow tract walls of control embryos (D-F). In Vangl2flox/flox; Isl1-Cre mutants, however, fibronectin can be seen surrounding cells throughout the outflow walls (L-N). O–T) Unlike the distal outflow tract where laminin distribution is abnormal in the Vangl2flox/flox; Isl1-Cre mutants, laminin is basally restricted in the proximal outflow tract in both controls (O-Q) and mutants (R-T). U) Diagrammatic representation of the outflow tract E9.5. Sections shown in panels A-N were taken from the distal outflow (upper box) whereas sections in panels O-T were from the proximal outflow (lower box). Scale bar = 20 µm (TIF) [file pgen.1004871.s009.tif]

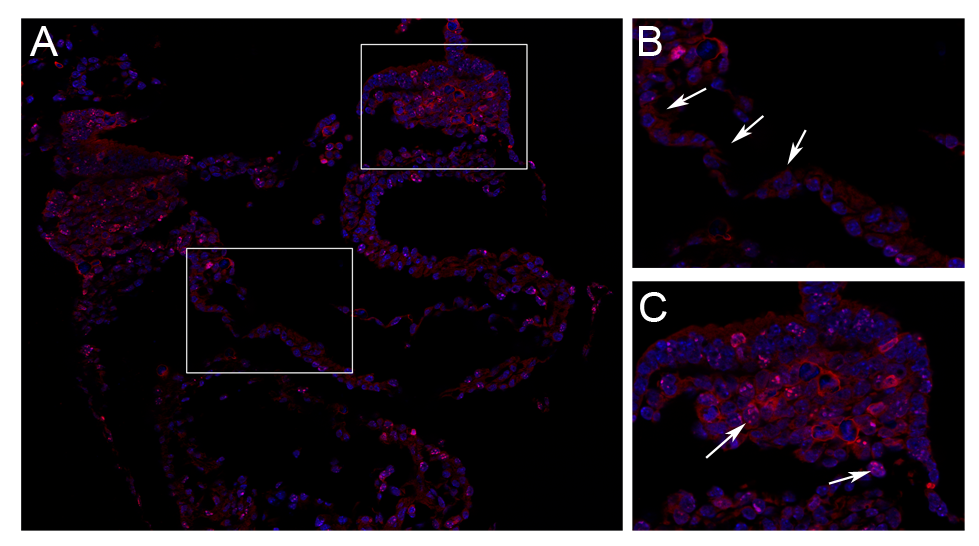

Supplement: S10 Fig — Absence of proliferation in cells in the distal outflow tract wall at E9.5. A,B) There is little or no proliferation in cells in the distal outflow tract wall (arrows in B) at E9.5. A,C) However, extensive proliferation is seen in the nearby pharyngeal arch (arrows in C). (TIF) [file pgen.1004871.s010.tif]

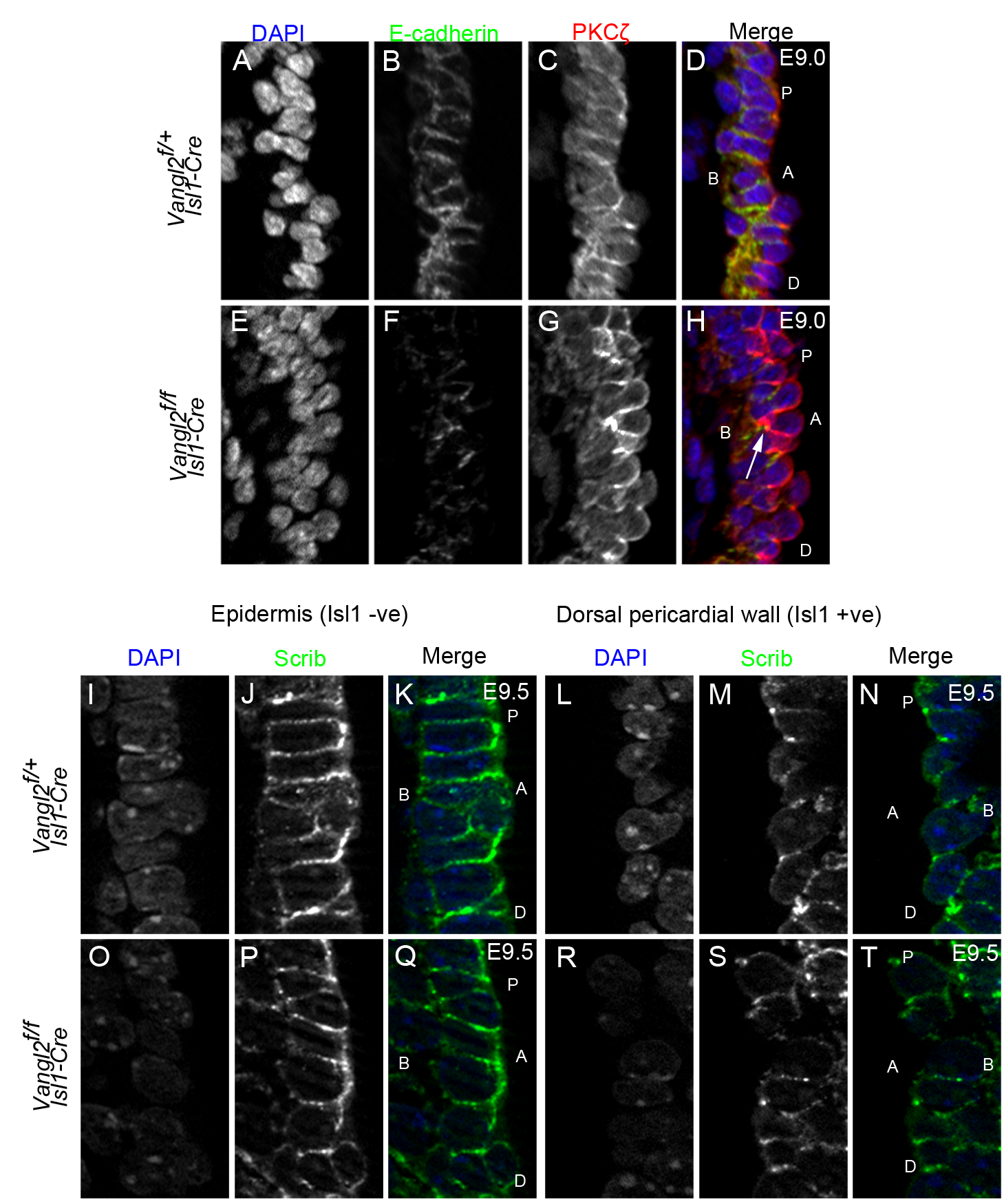

Supplement: S11 Fig — Disruption of epithelial organisation in the distal outflow tract of Vangl2flox/flox; Isl1-Cre embryos at E9.0. A–H) Similarly to at E9.5, E-cadherin and aPKCζ are mislocalised in the distal outflow tract of mutant embryos (E–H), compared with stage-matched littermates (A–D). Notably, aPKCζ is found basally (arrow in H) rather than apically in some cells in the mutant embryo. I–T) Scrib staining was normal in Vangl2flox/flox; Isl1-Cre mutants in the epidermis which does not express Isl1-Cre (O-Q, compare to I-K) and in the dorsal pericardial wall which is Isl1-Cre-positive (compare R–T with L–N). (TIF) [file pgen.1004871.s011.tif]
